# Arterial stiffness is associated with small and large fiber neuropathy: The Maastricht Study

**DOI:** 10.1097/HJH.0000000000004126

**Published:** 2025-09-26

**Authors:** Chidera Okoro, Carla J.H. van der Kallen, Sara B.A. Mokhtar, Tan Lai Zhou, Tos T.J.M. Berendschot, Annemarie Koster, Koen Reesink, Jos Reulen, Werner H. Mess, Carroll A.B. Webers, Abraham Kroon, Coen D.A. Stehouwer

**Affiliations:** a CARIM School for Cardiovascular Diseases, Maastricht University (UM); b Department of Internal Medicine, Maastricht University Medical Center^+^ (MUMC^+^); c MHeNS School of Mental Health and Neuroscience, UM; d University Eye Clinic Maastricht, MUMC+; e CAPHRI Care and Public Health Research Institute; f Department of Social Medicine, UM; g Department of Clinical Neurophysiology, MUMC+; h Department of Biomedical Engineering, UM, Maastricht, the Netherlands; i Department of Chronic Diseases and Metabolism (CHROMETA), KU Leuven, Belgium

**Keywords:** carotid–femoral pulse wave velocity, peripheral neuropathies, vascular stiffness

## Abstract

**Aim:**

To examine the association between arterial stiffness as determined by carotid–femoral pulse wave velocity (cfPWV) and neuropathy (retinal, corneal, and peripheral).

**Methods:**

We used population-based cross-sectional data from The Maastricht Study of *N* = 9188 participants (mean age 59.5 years, 50.2% women, 21% had type 2 diabetes) to study the associations between arterial stiffness and retinal nerve layer thickness, retinal sensitivity, corneal nerve measures, peripheral nerve conduction velocities, amplitudes, and vibration perception thresholds. We used linear regression analyses with adjustment for potential confounders and tested for interactions by sex and glucose metabolism status (prediabetes and type 2 diabetes vs. normal glucose metabolism), expressed as standardized betas.

**Results:**

After adjustment, greater arterial stiffness was associated with lower *Z* scores for retinal nerve layer thickness [−0.04 (−0.07 to −0.00]), mean retinal sensitivity [−0.06 (−0.09 to −0.03)], corneal nerve measures [−0.05 (−0.09 to −0.01)], peripheral nerve conduction velocities [−0.05 (−0.08 to −0.03)], and tibial nerve amplitude [−0,05 (−0.08 to −0.01)], and higher *Z* scores for mean peripheral vibration perception thresholds [0.06 (0.03–0.08)]. In general, directionally similar associations were observed for all individual components. However, there was no significant association between the *Z* score for peroneal nerve amplitude [0.00 (−0.04 to 0.04)] and the *Z* score for sural nerve amplitude [0.00 (−0.04 to 0.03)]. The results were similar in men and women as well as in people across different glucose metabolism strata.

**Conclusion:**

Our findings show that arterial stiffening is associated with both small-fiber and large-fiber neuropathies. Further longitudinal research is needed to clarify whether arterial stiffening is a direct cause of neuropathy.

## INTRODUCTION

Neuropathy, characterized by the gradual deterioration of the structure or functionality of neurons, affects millions of individuals and imposes medical and public health burdens on populations worldwide. Disorders such as peripheral and optic neuropathy are recognized as significant conditions that lead to peripheral nerve damage, vision loss, and morbidity [1,2]. Increased cardiovascular risk has been associated with neuropathy [3,4], we hypothesized that greater arterial stiffness, which is an important and independent cardiovascular risk factor [5], is a potential determinant of neuropathy.

Arterial stiffness, a hallmark of vascular aging, is caused by reduced arterial compliance and increased vascular resistance. It disrupts the normal blood flow throughout the body and affects various organs and tissues. In large peripheral nerves, arterial stiffness can potentially harm the vasa nervorum by affecting microcirculation [6], while in small nerves lacking the vasa nervorum, such as the corneal nerve, arterial stiffness may impair oxygen and nutrient delivery through endothelial dysfunction, inflammation, and local thrombosis, thus affecting diffusion from capillary networks in adjacent tissues [7] including the epineurium and perineurium [8].

To the best of our knowledge, no population-based study has investigated the association between arterial stiffness, that is, carotid-femoral pulse wave velocity (cfPWV), and degeneration in both smaller (ocular) and larger (peripheral) nerve fibers. One meta-analysis [9] showed an association between greater arterial stiffness and peripheral neuropathy; however, these studies were relatively small, used various methods to measure PWV, and included only individuals with diabetes [10–13]. In addition, others studies did not use the current gold standard as index for arterial stiffness [14,15].

To test our hypothesis, we investigated the associations between arterial stiffness, measured by cfPWV, and the retinal, corneal, peroneal, tibial, and sural nerves in a large population-based Maastricht Study.

## METHODS

### Study population and design

We used data from The Maastricht Study, a prospectively designed, population-based, observational cohort study. The rationale and methodology have been described previously [16] In brief, this study focuses on the etiology, pathophysiology, complications, and comorbidities of type 2 diabetes mellitus and is characterized by an extensive phenotyping approach. All individuals aged 40–75 years who lived in the southern part of the Netherlands were eligible for participation [16]. The present report includes cross-sectional data of *N* = 9188 participants who were included in the baseline survey between November 2010 and October 2020. The examinations of each participant were performed within a time window of 3 months (more details regarding corneal confocal microscopy measurements lag time are provided in the Supplemental Material). This study was approved by the Institutional Medical Ethical Committee (NL31329.068.10) and the Minister of Health, Welfare, and Sports of the Netherlands (Permit 131088-105234-PG). All the participants provided written informed consent [16].

### Assessment of carotid–femoral pulse wave velocity

Measurements were performed by trained vascular technicians in a dark, quiet, temperature-controlled room (21–23 °C), as described previously [17] and in Supplemental Methods. Briefly, cfPWV (in m/s) was determined according to international guidelines using applanation tonometry (SphygmoCor, Atcor Medical, Sydney, Australia) [18]. Pressure waveforms were determined at the right common carotid and right common femoral arteries. The difference in the time of pulse arrival from the R-wave of the ECG between the two sites (transit time) was determined by using the intersecting tangent algorithm. The pulse wave travel distance was calculated as 80% of the direct straight distance (measured using an infantometer) between the two arterial sites. The median of three consecutive cfPWV (defined as traveled distance/transit time) recordings was used in the analyses.

### Assessment of neuropathy

For the extended methodology, we refer to the Supplemental Methods.

#### Retinal nerve layer thickness

We assessed the average thickness of the retina of both eyes in the central macular area [Early Treatment Diabetic Retinopathy Study (ETDRS) sectors 1–5] using optical coherence tomography (Heidelberg Engineering, Heidelberg, Germany) with a fovea-centered macular volume scan (73 sections, 60 μm). We assessed the thickness of the following macular retinal layers: nerve fiber, ganglion cell, and inner plexiform layers. The average thickness of both eyes was used to calculate the thickness of the retinal layers.

#### Retinal sensitivity

We assessed retinal sensitivity of both eyes in the central and perimacular areas using a Heidelberg Edge Perimeter (Heidelberg Engineering, Heidelberg, Germany). In brief, light stimuli varying in strength between 0 and 35 dB were presented at 54 coordinates on the retina; at each coordinate, the threshold of visual perception was determined, and the results were averaged into ‘retinal sensitivity’.

#### Corneal nerve measures

Corneal confocal microscopy (Heidelberg Retina Tomograph III, Rostock Cornea Module, Heidelberg Engineering, Heidelberg, Germany) was used to image the corneal nerves in the left eye [19]. We used a U-Net-Based Convolutional Neural Network to automatically trace and analyze the following indices of corneal nerves: corneal nerve bifurcation density, corneal nerve density, corneal nerve length, and corneal nerve fractal dimension [20].

#### Peripheral nerve conduction velocities and amplitudes

Nerve conduction velocity (NCV) was measured in the peroneal and tibial motor nerves and the sural sensory nerve using Medelec Synergy electromyography (EMG) (V.15.0, Viasys Healthcare UK Ltd, Warwick, UK) with surface electrodes. Briefly, compound muscle action potential (CMAP) amplitudes (stimulated at the ankle), sural sensory nerve action potential (SNAP) amplitudes, and NCV of the peroneal, tibial, and sural nerves were measured.

#### Peripheral vibration perception thresholds

Peripheral vibration perception threshold was tested using a Horwell Neurothesiometer (Scientific Laboratory Supplies, Nottingham, UK). The vibration thresholds were tested three times at the distal phalanx of the hallux on both feet. The mean threshold was calculated for the left and right feet, and these, along with the mean of both feet, were used for the analyses.

### Assessment of covariates

We used fasting plasma glucose and 2-h postload glucose levels to assess glucose metabolism status according to the WHO 2006 criteria as normal glucose metabolism, prediabetes, type 2 diabetes, type 1 diabetes, or other types of diabetes. We assessed educational status (low, middle, or high), smoking status (never, current, or former), alcohol consumption (none, low, or high), prior cardiovascular disease, the Dutch Healthy Diet score, and income level using validated questionnaires [21,22]; assessed medication use via a medication interview; assessed waist circumference and office blood pressure as part of a physical examination; determined the total cholesterol to high-density lipoprotein ratio in fasting blood samples [21,23]; and assessed physical activity using an accelerometer [16,24,25]; estimated glomerular filtration rate (eGFR; in ml/min/1.73 m^2^) was calculated with the Chronic Kidney Disease Epidemiology equation based on serum creatinine; presence of albuminuria was defined as an average urinary albumin excretion greater than 30 mg per 24 h measured in two 24 h urine samples [16]; chronic kidney disease was defined as an eGFR less than 60 ml/min/1.73 m^2^ and (or) albuminuria. More details on the assessment of covariates are described in the Supplemental Methods.

### Statistical analyses

Continuous variables are reported as mean (SD) or median (25th and 75th percentiles), and categorical variables are expressed as percentages.

To simplify the analyses, we calculated *Z* scores for the retinal (inner plexiform layer, ganglion cell layer, and retinal nerve fiber layer), corneal (corneal nerve bifurcation density, corneal nerve density, corneal nerve length, and corneal nerve fractal dimension), peripheral nerve conduction velocities (peroneal, tibial, and sural nerve conduction velocities), and peripheral nerve amplitudes (peroneal CMAP, tibial CMAP, and sural SNAP), and averaged the two retinal sensitivity variables and the two peripheral vibration perception threshold outcomes. Next, the *Z* scores for each outcome were computed by averaging the individual Z scores. We checked whether all components exhibited directionally consistent associations with cfPWV, and this was the case.

We used multivariable linear regression analyses to investigate the association of the *Z* score for cfPWV with the *Z* scores for retinal nerve layer thickness, retinal sensitivity, corneal nerve measures, peripheral nerve conduction velocities and amplitudes, and peripheral vibration perception thresholds.

Model 1 presents crude results. Model 2 was adjusted for age, sex, educational status, glucose metabolism status (entered as dummy variables for prediabetes, and type 2 diabetes vs. normal glucose metabolism status), and office mean arterial pressure. We chose these variables because they are key potential confounders. Model 3 was, in addition to model 2, adjusted for waist circumference, alcohol consumption, smoking status, total cholesterol-to-high-density lipoprotein (HDL) cholesterol ratio, use of lipid-modifying medication, Dutch Healthy Diet score, and accelerometer-assessed physical activity. We adjusted for these covariates in model 3 because these factors may be additional potential confounders.

### Additional analyses

We performed several additional analyses. First, we investigated the association between cfPWV and the individual components of neuropathy outcomes. Second, we additionally adjusted for potential confounders that were not included in the main analyses because these covariates may not only be confounders but also mediators [history of cardiovascular disease, chronic kidney disease, use of diabetes medication, use of specific antihypertensive medications (ACE inhibitors, angiotensin receptor blockers, and aldosterone antagonists), and the use of other groups of antihypertensive medications]. Third, we substituted waist circumference with BMI, educational status with income level, glucose metabolism status with fasting plasma glucose and HbA1c, and office mean arterial pressure with 24-h ambulatory mean arterial pressure. Fourth, for corneal nerve measures, we further adjusted for corneal confocal microscopy lag times. Fifth, for peripheral nerve conduction velocities, we adjusted for height, as previous studies suggest that there is a strong inverse association between height and NCV [26]. Sixth, for both retinal nerve layer thickness and corneal nerve measures, we further adjusted for intraocular pressure. Seventh, in model 2, we additionally adjusted for heart rate. Eighth, for retinal sensitivity, we conducted additional analyses, excluding images with more than 15% false positives and more than 30% false negatives (*N* = 429). Finally, to compare the associations between cfPWV and age for each variable, we studied the association between age and composite scores of the outcomes.

We tested for interaction by sex, as associations may differ according to sex and glucose metabolism status due to the study design.

All analyses were performed using Statistical Package for Social Sciences version 25.0 (IBM SPSS, IBM Corp, Armonk, New York, USA). A *P* value less than 0.05 was considered statistically significant.

## RESULTS

### Selection and characteristics of the study population

**FIGURE 1:**
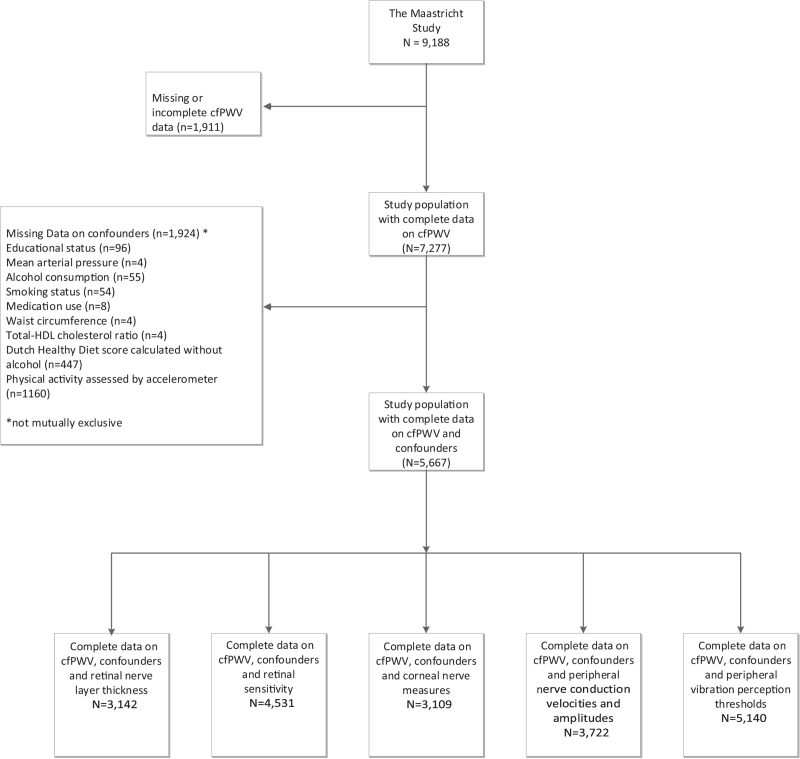
The selection of participants for inclusion in analyses. cfPWV, carotid–femoral pulse wave velocity; HDL, high-density lipoprotein.

Figure 1 presents an overview of the study-population selection process. We obtained complete data on cfPWV, covariates, and outcomes for 3142 participants for retinal nerve layer thickness, 4531 participants for retinal sensitivity, 3109 participants for corneal nerve measures, 3722 participants for peripheral nerve conduction velocities and amplitudes, and 5410 participants for peripheral vibration perception. Table 1 and Supplemental Tables S1–S4 show the general characteristics of the study population for retinal sensitivity, retinal nerve layer thickness, corneal nerve measures, peripheral nerve conduction velocities and amplitudes, and peripheral vibration perception thresholds, respectively. Overall, participants with lower composite neuropathy scores were older and had a worse cardiovascular risk profile.

**TABLE 1:** General study population characteristics according to tertiles of mean retinal sensitivity in the study population with complete data on carotid–femoral pulse wave velocity

Characteristic	Mean retinal sensitivity			
	Total study group (*N* = 4531)	Tertile 1 (low) (*N* = 1501)	Tertile2 (middle) (*N* = 1523)	Tertile 3 (high) (*N* = 1507)
Demographic characteristics				
Age (years)	59.95 ± 8.67	64.28 ± 7.95	59.90 ± 8.07	55.68 ± 7.78
Female [No. (%)]	2271 (50.1)	757 (50.4)	766 (50.3)	748 (49.6)
Educational level				
Low	1541 (34.0)	645 (43.0)	504 (33.1)	392 (26.0)
Middle	1220 (26.9)	353 (23.5)	410 (26.9)	457 (30.3)
High	1770 (39.1)	503 (33.5)	609 (40.0)	658 (43.7)
Cardiovascular risk factors				
Glucose metabolism status				
Normal glucose metabolism	2875 (63.5)	821 (54.7)	980 (64.3)	1074 (71.3)
Prediabetes	668 (14.7)	255 (17.0)	236 (15.5)	177 (11.7)
Type 2 diabetes	965 (21.3)	416 (27.7)	301 (19.8)	248 (16.5)
Type 1 and other type of diabetes	23 (0.5)	9 (0.6)	6 (0.4)	8 (0.5)
Mean arterial pressure (mmHg)	94.50 ± 11.28	94.92 ± 11.38	94.21 ± 11.04	94.36 ± 11.42
Waist circumference (cm)	94.44 ± 13.23	95.89 ± 13.20	94.25 ± 13.11	93.20 ± 13.26
Total/HDL cholesterol ratio	3.56 ± 1.18	3.51 ± 1.14	3.57 ± 1.23	3.60 ± 1.17
Use of lipid-modifying medication (yes vs. no)	1312 (29.0)	593 (39.5)	405 (26.6)	314 (20.8)
Lifestyle factors				
Alcohol consumption				
None	795 (17.5)	305 (20.3)	236 (15.5)	254 (16.9)
Low (women ≤ 7, men<=14)	2707 (59.7)	844 (56.2)	904 (59.4)	959 (63.6)
High (women > 7, men>14)	1029 (22.7)	352 (23.5)	383 (25.1)	294 (19.5)
Smoking status				
Never	1748 (38.6)	536 (35.7)	598 (39.3)	614 (40.7)
Former	2250 (49.7)	801 (53.4)	745 (48.9)	704 (46.7)
Current	533 (11.8)	164 (10.9)	180 (11.8)	189 (12.5)
Determinant				
Carotid–femoral pulse wave velocity (m/s)	8.93 ± 2.10	9.50 ± 2.34	8.88 ± 2.04	8.41 ± 1.75
Outcomes				
Retinal sensitivity				
Retinal sensitivity of left eye	27.50 ± 2.18	25.71 ± 2.80	27.77 ± 0.62	29.00 ± 0.71
Retinal sensitivity of right eye	27.66 ± 2.28	25.75 ± 2.93	28.02 ± 0.63	29.20 ± 0.69

Data are presented as mean ± standard deviation, median [interquartile range] or number (%). HDL, high-density lipoprotein; SD, standard deviation.

### Association with retinal nerve layer thickness

According to model 3, greater cfPWV was significantly associated with a lower composite *Z* score for retinal nerve layer thickness [standardized beta coefficient (st*β*) (95% CI) −0.04 (−0.07 to −0.00)].

### Association with retinal sensitivity

According to model 3, greater cfPWV was significantly associated with a lower *Z* score for mean retinal sensitivity [st*β* (95% CI) −0.06 (−0.09 to −0.03)].

### Association with corneal nerve measures

According to model 3, greater cfPWV was significantly associated with a lower composite *Z* score for corneal nerve measures [st*β* (95% CI) −0.05 (−0.09 to −0.01)].

### Association with peripheral nerve conduction velocities and amplitudes

According to model 3, greater cfPWV was significantly associated with a lower composite *Z* score for peripheral nerve conduction velocities [st*β* (95% CI) −0.05 (−0.08 to −0.02)] and lower tibial nerve amplitude [st*β* (95% CI) −0,05 (−0.08 to −0.01)]. However, there were no significant associations with the peroneal nerve amplitude [st*β* (95% CI) 0.00 (−0.04 to 0.04); and sural nerve amplitude [st*β* (95% CI) 0.00 (−0.04 to 0.03)].

### Association with peripheral vibration perception thresholds

**FIGURE 2:**
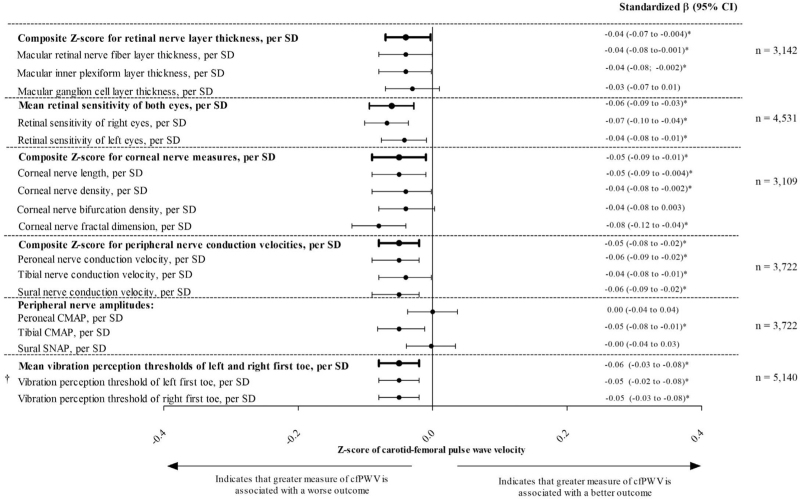
Associations of carotid–femoral pulse wave velocity with retinal nerve layer thickness, retinal sensitivity, corneal nerve measures, peripheral nerve conduction velocities, amplitudes, and peripheral vibration perception thresholds. Standardized regression coefficients (*β*) represent the differences in retinal nerve layer thickness, retinal sensitivity, corneal nerve measures, peripheral nerve conduction velocities, and peripheral vibration perception thresholds in SD per SD of greater arterial stiffness. One SD corresponds with 4.1 μm macular retinal nerve fiber layer thickness, 4.6 μm macular ganglion cell layer thickness, 16.7 μm total retinal layer thickness, 3.2 μm macular inner plexiform layer thickness, 0.8 (unit-less) for composite *Z* score for retinal nerve layer thickness measures, 2 dB for retinal sensitivity, 0.9 (unit-less) for composite *Z* score for corneal nerve measures, 41.5 number of branches/mm^2^ for corneal nerve bifurcation density, 26.2 number of main fibers/mm^2^ for corneal nerve density, 0.1 (unit-less) for corneal nerve fractal dimension, 5.1 mm/mm^2^ for corneal nerve length, 0.8 (unit-less) for composite *Z* score for peripheral nerve conduction velocity measures, 4.4 m/s for peroneal nerve conduction velocity, 4.5 m/s for tibial nerve conduction velocity, 6 m/s for sural nerve conduction velocity, 2 mV for peroneal nerve CMAP amplitude, 4.0 mV for tibial nerve CMAP amplitude, 6.4 μV for sural nerve SNAP amplitude, 8 volts for vibration perception threshold, and 2.1 m/s for carotid–femoral pulse wave velocity. Variables entered in the model: crude + adjusted for age, sex, glucose metabolism status, educational level, mean arterial pressure, waist circumference, alcohol consumption status, smoking status, total cholesterol to HDL cholesterol ratio, use of lipid-modifying medication, Dutch Health Diet score, and accelerometer-assessed physical activity. ^*^Indicates statistical significance (*P* < 0.05). ^†^Vibration perception threshold was inversed (multiplied by −1) so that a lower VPT (more negative) represented a worse outcome. μV, microvolts; CI, confidence interval; CMAP, compound muscle action potential; dB, decibel; HDL, high-density lipoprotein; *N*, population sample size; SD, standard deviation; SNAP, sensory nerve action potential; VPT, vibration perception threshold.

According to model 3, greater cfPWV was significantly associated with an increased *Z* score for the mean peripheral vibration perception threshold [st*β* (95% CI) 0.06 (0.03–0.08); Fig. 2].

### Additional analyses

The results were largely similar when we separately analyzed the association between cfPWV and the individual components of neuropathy outcomes (Table S5).

Associations did not materially change after additional adjustment for history of cardiovascular disease, chronic kidney disease, use of diabetes medication, use of specific antihypertensive medications, heart rate, and after waist circumference was replaced with BMI, glucose metabolism status with fasting plasma glucose and HbA1c, educational level with income level, or office mean arterial pressure by 24-h ambulatory mean arterial pressure (Tables S7-S8). Similar results were observed after adjusting for corneal nerve measures lag time, height in peripheral NCV, and intraocular pressure for both retinal nerve layer thickness and corneal nerve measures (Table S8). For retinal sensitivity, we obtained similar results when we excluded images that did not meet the quality criteria (Table S9).

Associations were not modified by glucose metabolism status or sex (Table S10).

When associations with cfPWV were compared with those of age, we observed that the associations of 2 m/s greater cfPWV were equivalent to 4, 2, 5, 3, 2 and 1 years of additional aging in retinal nerve layer thickness, retinal sensitivity, corneal nerve measures, peripheral nerve conduction velocities, tibial nerve amplitude and peripheral vibration perception thresholds, respectively (Tables S11-S12).

## DISCUSSION

This population-based study has two main findings. First, greater arterial stiffness, as determined by cfPWV, was significantly associated with lower small nerve fiber thickness and function, as shown by the lower composite *Z* scores for retinal nerve layer thickness, retinal sensitivity, and corneal nerve measures. Second, greater arterial stiffness was significantly associated with impaired large nerve fiber function, as shown by lower composite *Z* scores for peripheral nerve conduction velocities and lower *Z* scores for tibial nerve amplitude, along with a higher *Z* score for peripheral vibration perception thresholds. Importantly, these associations were independent of confounders, such as diabetes, hypertension, renal insufficiency, and prior cardiovascular disease. The strength of these associations was that each SD greater arterial stiffness was equivalent to approximately 1–5 years of additional aging in nerve functioning and structure, respectively.

To our knowledge, this is the first large population-based study to demonstrate that arterial stiffness is consistently associated with both ocular (small) and peripheral (large) fiber neuropathies. Specifically, our findings showed significant associations between arterial stiffness and individual components of retinal nerve layer thickness, retinal sensitivity, corneal nerve measures, peripheral nerve conduction velocities, amplitudes, and peripheral vibration perception thresholds. These findings provide new insights into the potential link between arterial stiffness and neuropathic process of both small and large nerve fibers.

Our findings are consistent with those of previous studies [10–13,27–29], suggesting a relationship between arterial stiffness and neuropathy. However, these studies were either relatively small [10–13] or did not use cfPWV [15,27]. Huang *et al.* [15] found no association between arterial stiffness and retinal neuropathy in a large population-based study. However, arterial stiffness was calculated as the arterial stiffness index using pulse waveforms from the finger, which has only been moderately correlated with cfPWV [30]. This may explain the contrasting results from the present study, as we used cfPWV, which is widely regarded as the gold standard [14] for evaluating arterial stiffness.

We observed that greater arterial stiffness was associated with lower composite scores for retinal, corneal, and peripheral nerve functions. The advantage of using composite scores is that the impact of technical and biological variability is reduced. Importantly, associations with the individual components of the scores showed directionally similar results (Table S5).

The mechanisms linking arterial stiffness and neuropathy remain incompletely understood. A plausible explanation involves microvascular dysfunction, which may arise due to mechanical stress exerted by increased pulsatile energy on endothelial cells [31]. Endothelial dysfunction involves impaired regulation of vasodilation and vasoconstriction as well as impairment of antithrombotic and anti-inflammatory properties, setting the stage [32] for a chronic inflammatory response [33,34], creating a self-perpetuating cycle that further exacerbates endothelial dysfunction. Additionally, localized thrombosis within the microcirculation [35] may obstruct blood flow, leading to inadequate nutrient delivery to the nerves while simultaneously promoting heightened permeability of adjacent blood vessels. The latter, in turn, may allow infiltration of inflammatory cells and molecules into nerve tissue. Interestingly, we found that greater arterial stiffness was associated with lower peripheral nerve conduction velocities but was only partially associated with lower peripheral nerve amplitudes. This may be because the process of demyelination (i.e. lower peripheral NCV) occurs first, while axonal loss (i.e. lower peripheral nerve amplitude) manifests at a more advanced stage, as has been reported in previous studies [36,37].

Our findings have clinical implications. The observed associations between greater arterial stiffness and both small-fiber and large-fiber neuropathy highlight the importance of vascular health in patients with neuropathy. Clinicians may consider testing for neuropathy in patients with worse cardiovascular health and, conversely, optimize cardiovascular risk management when treating patients with established neuropathy. Further interventional research is needed to investigate whether such interventions are beneficial.

Our study has several strengths. First, the inclusion of a large population-based cohort, notably oversampled for individuals with type 2 diabetes, allowed us to investigate our hypothesis specifically within this population. Second, we used cfPWV, which is considered the gold standard for assessing arterial stiffness. Third, we adjusted for mean blood pressure derived from 24-h blood pressure monitoring. Fourth, our analysis involved comprehensive adjustments for a wide range of potential confounding variables, and our results were robust after many additional adjustments.

The present study has some limitations. First, due to the cross-sectional design, we cannot draw conclusions about causality. Reverse causality is also possible, as previous research suggests that diabetic neuropathy may contribute to arterial stiffening through mechanisms, such as reduced vascular innervation and increased arterial calcification [38]. Second, residual confounding cannot be excluded. We did not account for potential shared pathophysiological mechanisms such as endothelial dysfunction, low-grade inflammation, and oxidative stress, which may underlie both neuropathy [39] and arterial stiffness [40], which may have overestimated our results. Third, our study primarily focused on a Caucasian population aged 40–75 years. Consequently, the generalizability of our findings to other demographic groups and age ranges remains uncertain. Further research is required to investigate these associations in diverse ethnic populations and across different age groups.

In conclusion, the present population-based study demonstrated that greater arterial stiffness, determined by cfPWV, was associated with ocular (small) and peripheral (large) fiber neuropathy. These results were independent of important confounders, such as diabetes, hypertension, renal insufficiency, and prior cardiovascular disease. Future longitudinal research is needed to clarify whether arterial stiffening is a direct cause of neuropathy.

## ACKNOWLEDGEMENTS

The authors would like to acknowledge the ZIO foundation (Vereniging Regionale HuisartsenZorg Heuvelland) for their contribution to The Maastricht Study. The researchers are indebted to the participants for their willingness to participate in the study.

Funding sources: this study was supported by the European Regional Development Fund via OP-Zuid, the Province of Limburg, the Dutch Ministry of Economic Affairs (grant 31O.041), Stichting De Weijerhorst (Maastricht, the Netherlands), the Pearl String Initiative Diabetes (Amsterdam, the Netherlands), the Cardiovascular Center (CVC, Maastricht, the Netherlands), CARIM School for Cardiovascular Diseases (Maastricht, the Netherlands), CAPHRI School for Public Health and Primary Care (Maastricht, the Netherlands), NUTRIM School for Nutrition and Translational Research in Metabolism (Maastricht, the Netherlands), Stichting Annadal (Maastricht, the Netherlands), Health Foundation Limburg (Maastricht, the Netherlands), Perimed (Järfälla, Sweden), and by unrestricted grants from Janssen-Cilag B.V. (Tilburg, the Netherlands), Novo Nordisk Farma B.V. (Alphen aan den Rijn, the Netherlands), and Sanofi-Aventis Netherlands B.V. (Gouda, the Netherlands).

Data availability: data are available from The Maastricht Study for any researcher who meets the criteria for access to confidential data; the corresponding author may be contacted to request data.

### Conflicts of interest

There are no conflicts of interest.

## Supplementary Material

Supplemental Digital Content
