# Supplementary material for: Arterial stiffness is associated with small and large fiber neuropathy: The Maastricht Study
Source: J Hypertens. 2025 Sep 26;43(12):1945–52. doi: 10.1097/HJH.0000000000004126 (PMC12582623; doi:10.1097/HJH.0000000000004126)
Supplement: Supplemental Digital Content [file jhype-43-1945-s001.docx]

**Supplemental Material**

**Content**

Supplemental Methods

Supplemental Tables

- **Supplemental Table S1** Additional general study population characteristics based on tertiles of composite Z-score for retinal nerve layer thickness in the study population with complete data on carotid-femoral pulse wave velocity.
- **Supplemental Table S2** Additional general study population characteristics based on tertiles of composite Z-score for corneal nerve measures with complete data on carotid femoral pulse wave velocity.
- **Supplemental Table S3** Additional general study population characteristics based on tertiles of composite Z-score for peripheral nerve conduction velocities and amplitudes with complete data on carotid femoral pulse wave velocity.
- **Supplemental Table S4** Additional general study population characteristics based on tertiles of mean peripheral vibration perception thresholds in the study population with complete data on carotid-femoral pulse wave velocity.
- **Supplemental Table S5** Associations of carotid-femoral pulse wave velocity with both individual components and composite z-scores for retinal nerve layer thickness, retinal sensitivity, corneal nerve measures, peripheral nerve conduction velocities, amplitudes and peripheral vibration perception threshold.
- **Supplemental Table S6** Associations of carotid-femoral pulse wave velocity with composite z-scores for retinal nerve layer thickness, retinal sensitivity, corneal nerve measures, peripheral nerve conduction velocities and peripheral vibration perception threshold additionally adjusted for history of cardiovascular disease, chronic kidney disease, use of diabetes medication (model 4A); use of specific anti-hypertensive medications (ACE inhibitors, angiotensin receptor blockers (ARBs), aldosterone antagonists (model 4B); and the use of other groups of anti-hypertensive medications (model 4C).
- **Supplemental Table S7** Associations of carotid-femoral pulse wave velocity with peripheral nerve amplitudes, additionally adjusted for history of cardiovascular disease, chronic kidney disease, use of diabetes medication (model 4A); use of specific anti-hypertensive medications (ACE inhibitors, angiotensin receptor blockers (ARBs), aldosterone antagonists (model 4B); and the use of other groups of anti-hypertensive medications (model 4C); where waist circumference was replaced with BMI (model 5A), or glucose metabolism status was replaced with fasting plasma glucose (model 5B), with HbA1c (model 5C), or educational level was replaced with income level (model 5D) and office mean arterial pressure was replaced by 24-hour ambulatory mean arterial pressure (model 5E).
- **Supplemental Table S8** Associations of carotid-femoral pulse wave velocity with composite Z-score for retinal nerve layer thickness, retinal sensitivity, corneal nerve measures, peripheral nerve conduction velocities and vibration perception threshold where waist circumference was replaced with BMI (model 5A), or glucose metabolism status was replaced with fasting plasma glucose (model 5B), with HbA1c (model 5C), or educational level was replaced with income level (model 5D) and office mean arterial pressure was replaced by 24-hour ambulatory mean arterial pressure (model 5E), with corneal nerve measures additionally adjusted for CCM lag time(model 6), peripheral nerve conduction velocities additionally adjusted for height(model 7), and finally all composite measures additionally adjusted for heart rate and retinal nerve layer thickness and corneal nerve measures additionally adjusted for intraocular pressure.
- **Supplemental Table S9** Associations of carotid-femoral pulse wave velocity with retinal sensitivity excluding images with percentage false positives >15% and percentage false negatives >30%.
- **Supplemental Table S10** P-values for interaction by sex, prediabetes, and type 2 diabetes in the associations of carotid-femoral pulse wave velocity with retinal nerve layer thickness, retinal sensitivity, corneal nerve measures, peripheral nerve conduction velocities and peripheral vibration perception threshold.
- **Supplemental Table S11** Associations of age with composite Z-score for retinal nerve layer thickness, retinal sensitivity, corneal nerve measures, peripheral nerve conduction velocities and peripheral vibration perception threshold.
- **Supplemental Table S12** Associations of age with peripheral nerve amplitudes.

**Study population and design**

We used data from The Maastricht Study, a prospectively designed, population-based observational cohort study. The rationale and methodology have been described previously^(1)^. In brief, the study focuses on the etiology, pathophysiology, complications, and comorbidities of type 2 diabetes mellitus and is characterized by an extensive phenotyping approach. Eligible for participation were all individuals aged between 40 and 75 years and living in the southern part of the Netherlands. Participants were recruited through mass media campaigns and from the municipal registries and the regional Diabetes Patient Registry via mailings. Recruitment was stratified according to known type 2 diabetes status, with an oversampling of individuals with type 2 diabetes, for reasons of efficiency.^(1)^ The present report includes cross-sectional data of N= 9187 participants who were included in the baseline survey between November 2010 and December 2020. The examinations of each participant were performed within a time window of three months*.* Corneal confocal microscopy measurements were performed from April 2013 until January 2019. People who participated in The Maastricht Study before the start of corneal confocal microscopy measurement (i.e., before April 2013) were re-invited (n=974) (‘catch-up visit’). For these participants, there was a median time interval (‘visit interval’) of 5.2 years between corneal confocal microscopy measurements and all other measurements. The study has been approved by the institutional medical ethical committee (NL31329.068.10) and the Minister of Health, Welfare, and Sports of the Netherlands (Permit 131088-105234-PG). All participants gave written informed consent.^(1)^

**Assessment of carotid-femoral pulse wave velocity** (main determinant)

All measurements were done by trained vascular technicians unaware of the participants’ clinical or diabetes mellitus status, in a dark, quiet, temperature-controlled room (21°C–23°C), as described previously.^(2, 3)^ Participants were asked to refrain from smoking and drinking coffee, tea or alcoholic beverages for three hours before the study. Participants were allowed to have a light meal (breakfast or lunch). All measurements were performed in the supine position after ten minutes of rest. Talking or sleeping was not allowed during the examination. During the vascular measurements (≈45 minutes), brachial systolic, diastolic, and mean arterial pressure were determined every five minutes with an oscillometric device (Accutorr Plus, Datascope Inc, Montvale, NJ). The mean arterial pressure and heart rate during these measurements were used in the statistical analysis. A three-lead ECG was recorded continuously during the measurements to facilitate automatic signal processing. Carotid-femoral pulse wave velocity (cfPWV; in m/s) was determined according to international guidelines with the use of applanation tonometry (SphygmoCor, Atcor Medical, Sydney, Australia).^(4)^ Pressure waveforms were determined at the right common carotid arteries and right common femoral arteries. Difference in the time of pulse arrival from the R-wave of the ECG between the two sites (transit time) was determined with the intersecting tangents algorithm. The pulse wave travel distance was calculated as 80% of the direct straight distance (measured with an infantometer) between the two arterial sites. The median of three consecutive cfPWV (defined as traveled distance/transit time) recordings was used in the analyses.

**Assessment of outcomes**

*Retinal nerve layer thickness*

We assessed the average thickness of the retina of both eyes in the central macular area (Early Treatment Diabetic Retinopathy Study [ETDRS] sectors 1-5) with optical coherence tomography (OCT; Heidelberg Engineering, Heidelberg, Germany) using a fovea-centered macular volume scan (73 sections, 60 μm). We assessed the thickness of the following macular retinal layers: nerve fiber layer thickness; ganglion cell layer; and inner plexiform layer. We used the average thickness of both eyes to calculate the thickness of retinal layers.

We determined the thickness of individual retinal layers as follows. First, we exported data on individual voxels from the optical coherence tomography device using custom software from Heidelberg Engineering. Second, using MATLAB (MATLAB and Statistics Toolbox Release 2012b, The MathWorks, Inc., Natick, USA) we calculated the thickness of individual retinal layers per voxel. Third, we exported data on the thickness of individual retinal layers for voxels located in sectors 1-5 of the ETDRS grid and per eye summarized these data into mean thickness for all individual retinal layers. With the presently used software the thickness of individual retinal layers could only be determined if a minimum of 72 out of 73 slices were correctly outlined.

To check whether a more strict selection of macular optical coherence tomography images would impact our results, we graded a subset of macular optical coherence tomography scans (from ~2,500 participants) for the presence of measurement errors, as previously described.^(5)^ Measurement errors were defined as: incomplete scan (i.e. <73 slices); incorrect centering on the fovea; poor outlining of the retina; technical problem of the optical coherence tomography device visible; and poor image quality (i.e. signal-to-noise ratio [dB]<15)^(6)^ . Overall >95% of retinal scans were of sufficient quality and exclusion of scans of insufficient quality did not affect the results (data not shown). Hence, we did not exclude any images for the main analyses.

*Retinal sensitivity*

We assessed retinal sensitivity of both eyes in the central and perimacular area with the Heidelberg Edge Perimeter (Heidelberg Engineering, Heidelberg, Germany). In brief, light stimuli varying in strength between 0 and 35 decibels were presented at 54 coordinates on the retina; at each coordinate the threshold of visual perception (i.e., the threshold at which the weakest presented visual stimulus could be perceived) was determined; and results were averaged into ‘retinal sensitivity’. We conducted a grading process wherein we considered images with a maximum false positive rate of 15% and a maximum false negative rate of 30%. The intra-observer reliability for the assessment of the retinal sensitivity was 0.95^(7)^.

*Corneal nerve measures*

We used corneal confocal microscopy (Heidelberg Retina Tomograph III, Rostock cornea module, Heidelberg Engineering, Heidelberg, Germany) to image corneal nerves in the left eye.^(8)^ Before the measurement, we anesthetized both eyes with topical oxybuprocaine hydrochloride 0.4 mg/ml eye drops (Minims^®^, Bausch & Lomb; France), and, to ensure optimal contact between the cornea and the applanating cap, we wetted both eyes with carbomer 2 mg/g eye gel (Vidsic^®^, Bausch & Lomb; Belgium). Individuals who had a corneal transplant or had a corneal infection of the left eye were excluded from the measurement. Participants were instructed to fixate on a white light throughout the scan. Trained research assistants imaged the subbasal nerve plexus layer in different corneal regions (central and peripheral regions (i.e., at or above the inferior whorl)). We aimed to obtain centrally focused corneal nerve scans but accepted all images.

We assessed multiple recordings of 400 × 400 μm (384 × 384 pixels, 8 bit) assembled using a composite algorithm implemented in the HRT3 user interface (Heidelberg Engineering, Heidelberg, Germany), as previously described. ^(8)^ Real-time mapping was performed on an area up to 1600 × 1600 µm (1536 × 1536 pixels, 8 bit).

We used U-Net-Based Convolutional Neural Network to fully automatically trace and analyze the following indices of corneal nerves:^(9)^ corneal nerve bifurcation density (number of bifurcation points [branching points] per mm^2^), corneal nerve density (total number of corneal nerve fibers [including both main fibers and branches] per mm^2^, with ‘main nerve fibers’ referring to the largest and most prominent nerve fibers), corneal nerve length (total length of corneal nerve fibers in mm, both main and branches, per mm^2^) and corneal nerve fractal dimension (quantification of the nerve structure complexity). The intra-class and inter-observer correlation coefficients, both indices of reliability, were ≥ 0.97 and ≥ 0.89, respectively.^(10)^

*Peripheral nerve conduction velocities and amplitudes*

Nerve conduction velocity was measured in the peroneal and tibial motor nerves and the sural sensory nerve using a Medelec Synergy electromyography (EMG) apparatus (V.15.0, Viasys Healthcare UK Ltd, Warwick, UK) using surface electrodes. Before testing, feet and lower legs were heated up in warm water (38°C) for a minimum duration of ten minutes to ensure that skin temperature (measured on the dorsal surface of the foot) was >32°C. The three nerves were examined at supramaximal stimulation. The peroneal nerve was assessed on the right leg, at the extensor digitorum brevis muscle with stimulations at the ankle (seven cm proximal from the recording site), below and above the fibular head. Peroneal motor nerve conduction velocity was measured from below fibular head to ankle. Tibial motor nerve conduction velocity was measured on the left leg at the abductor hallucis muscle with stimulations at the ankle (nine cm proximal from the recording site) and in the popliteal fossa. Sural sensory nerve conduction velocity was measured antidromically on the left leg between the lateral malleolus and the Achilles tendon while stimulating 12 cm proximal to the recording site. For determination of the nerve conduction velocity the time to onset of the motor and sensory potential was used.

Variables analyzed were compound muscle action potential (CMAP) amplitudes (stimulated at the ankle), sural sensory nerve action potential (SNAP) amplitude, nerve conduction velocities (NCV) of the peroneal, tibial and sural nerves.

*Peripheral vibration perception thresholds*

Peripheral vibration perception threshold was tested by use of a Horwell Neurothesiometer (Scientific Laboratory Supplies, Nottingham, UK). Vibration thresholds were tested three times at the distal phalanx of the hallux on both feet. Mean threshold was calculated for the left and right foot, and these, along with the mean of both feet, were used for the analyses.

**Assessment of covariates**

We determined glucose metabolism status (normal glucose metabolism, prediabetes, type 2 diabetes and other types of diabetes) based on a 75-gram oral glucose tolerance test and use of glucose-lowering medication according to the World Health Organization 2006 criteria^(11)^. Fasting plasma glucose (mmol/L) and haemoglobin A1c (mmol/mol;%) were determined in venous plasma samples collected after an overnight fast. Education level (‘educational status’) was classified into three groups: low (none, primary or lower vocational education only), medium (intermediate general secondary, intermediate vocational or higher general secondary education), and high (higher vocational education or university level of education). As described previously, we used questionnaires to assess income level ^(12)^ and cardiovascular disease history (defined as a history of any of the following conditions: myocardial infarction, cerebrovascular infarction or hemorrhage, and percutaneous artery angioplasty of, or vascular surgery on, the coronary, abdominal, peripheral or carotid arteries). Estimated glomerular filtration rate (eGFR; in mL/min/1.73 m^2^) was calculated with the Chronic Kidney Disease Epidemiology equation based on serum creatinine. Presence of albuminuria was defined as an average urinary albumin excretion >30mg per 24 hours measured in two 24-hour urine samples as described previously^16^. Chronic kidney disease was defined as an estimated glomerular filtration rate (eGFR) below 60 ml/min/1.73m^2^ and (or) albuminuria. Office blood pressure was measured using an oscillometric device^19^. Systolic and diastolic blood pressures were recorded in millimeters of mercury (mm Hg). Hypertension was defined as an office systolic blood pressure of ≥140 mm Hg and/or an office diastolic blood pressure of ≥90 mm Hg, or if the use of antihypertensive drugs was reported. Mean arterial pressure (MAP) was calculated using the formula: MAP = diastolic blood pressure + 1/3 (systolic blood pressure - diastolic blood pressure). Assessed weight, height, and waist circumference during a physical examination; calculated body mass index (BMI; in kg/m^2^) based on body weight and height; measured office and 24-hour ambulatory blood pressure (in mm Hg);Alcohol consumption was classified as non-consumer, low-consumer (≤seven alcoholic drinks/week for women; ≤14 alcoholic drinks/week for men), or high-consumer (>seven alcoholic drinks/week for women; >14 alcohol drinks/week for men). We determined age, sex, smoking status (never, former, current), medication use, waist circumference, total/high-density lipoprotein (HDL) cholesterol ratio, triglycerides, and accelerometer-assessed physical activity^(1, 13, 14)^.  We used a validated food frequency questionnaire to assess the Dutch Healthy Diet score^(15, 16)^.

**References**

1. Schram MT, Sep SJS, van der Kallen CJ, Dagnelie PC, Koster A, Schaper N, et al. The Maastricht Study: an extensive phenotyping study on determinants of type 2 diabetes, its complications and its comorbidities. European Journal of Epidemiology. 2014;29(6):439-51.

2. Hermeling E RK, Kornmann LM, Reneman RS, Hoeks AP. The dicrotic notch as alternative time-reference point to measure local pulse wave velocity in the carotid artery by means of ultrasonography. J Hypertens. 2009;27:2028–35.

3. Willekes C, Hoeks AP, Bots ML, Brands PJ, Willigers JM, Reneman RS. Evaluation of off-line automated intima-media thickness detection of the common carotid artery based on M-line signal processing. Ultrasound Med Biol. 1999;25(1):57-64.

4. Van Bortel LM, Laurent S, Boutouyrie P, Chowienczyk P, Cruickshank JK, De Backer T, et al. Expert consensus document on the measurement of aortic stiffness in daily practice using carotid-femoral pulse wave velocity. J Hypertens. 2012;30(3):445-8.

5. De Clerck EEB, Schouten J, Berendschot T, Goezinne F, Dagnelie PC, Schaper NC, et al. Macular thinning in prediabetes or type 2 diabetes without diabetic retinopathy: the Maastricht Study. Acta Ophthalmol. 2018;96(2):174-82.

6. van der Heide FCT, Steens ILM, Limmen B, Mokhtar S, van Boxtel MPJ, Schram MT, et al. Thinner inner retinal layers are associated with lower cognitive performance, lower brain volume, and altered white matter network structure-The Maastricht Study. Alzheimers Dement. 2024;20(1):316-29.

7. Cui QN, Gogt P, Lam JM, Siraj S, Hark LA, Myers JS, et al. Validation and reproducibility of the Heidelberg Edge Perimeter in the detection of glaucomatous visual field defects. Int J Ophthalmol. 2019;12(4):577-81.

8. Allgeier S, Zhivov A, Eberle F, Koehler B, Maier S, Bretthauer G, et al. Image Reconstruction of the Subbasal Nerve Plexus with In Vivo Confocal Microscopy. Investigative Ophthalmology & Visual Science. 2011;52(9):5022-8.

9. Colonna A, Scarpa F, Ruggeri A. Segmentation of Corneal Nerves Using a U-Net-Based Convolutional Neural Network: First International Workshop, COMPAY 2018, and 5th International Workshop, OMIA 2018, Held in Conjunction with MICCAI 2018, Granada, Spain, September 16 - 20, 2018, Proceedings. 2018. p. 185-92.

10. De Clerck EEB, Schouten JSAG, Berendschot TTJM, Koolschijn RS, Nuijts RMMA, Schram MT, et al. Reduced corneal nerve fibre length in prediabetes and type 2 diabetes: The Maastricht Study. Acta Ophthalmologica. 2020;98(5):485-91.

11. van der Heide FCT, Zhou TL, Henry RMA, Houben A, Kroon AA, Dagnelie PC, et al. Carotid stiffness is associated with retinal microvascular dysfunction-The Maastricht study. Microcirculation. 2021;28(6):e12702.

12. Qi Y, Koster A, van Boxtel M, Köhler S, Schram M, Schaper N, et al. Adulthood Socioeconomic Position and Type 2 Diabetes Mellitus-A Comparison of Education, Occupation, Income, and Material Deprivation: The Maastricht Study. Int J Environ Res Public Health. 2019;16(8).

13. Sörensen BM, van der Heide FCT, Houben A, Koster A, T TJMB, J SAGS, et al. Higher levels of daily physical activity are associated with better skin microvascular function in type 2 diabetes-The Maastricht Study. Microcirculation. 2020;27(4):e12611.

14. Martens RJH, Houben A, Kooman JP, Berendschot T, Dagnelie PC, van der Kallen CJH, et al. Microvascular endothelial dysfunction is associated with albuminuria: the Maastricht Study. J Hypertens. 2018;36(5):1178-87.

15. van Dongen MC, Wijckmans-Duysens NEG, den Biggelaar LJ, Ocké MC, Meijboom S, Brants HA, et al. The Maastricht FFQ: Development and validation of a comprehensive food frequency questionnaire for the Maastricht study. Nutrition. 2019;62:39-46.

16. Looman M, Feskens EJ, de Rijk M, Meijboom S, Biesbroek S, Temme EH, et al. Development and evaluation of the Dutch Healthy Diet index 2015. Public Health Nutr. 2017;20(13):2289-99.

**Table S1 Additional general study population characteristics based on tertiles of composite Z-score for retinal nerve layer thickness in the study population with complete data on carotid-femoral pulse wave velocity.**

| **Characteristic** | | | **Composite Z-score for Retinal nerve layer thickness** | | | | |  |
| --- | --- | --- | --- | --- | --- | --- | --- | --- |
|  |  |  | Total study group  (N =3142) | Tertile 1 (low)  (N = 1047) | | Tertile2 (middle)  (N = 1048) | Tertile 3 (high)  (N = 1047) |  |
| **Demographic characteristics** | | |  |  | |  |  |  |
| Age (years) | | | 60.25 ± 8.44 | 62.22 ± 8.38 | | 59.67 ± 8.40 | 58.85 ± 8.17 |  |
| Female, No. (%) | | | 1618(51.5) | 553(52.8) | | 573(54.7) | 492(47.0) |  |
| Educational level | | |  |  | |  |  |  |
| Low | | | 1101(35.0) | 396(37.8) | | 377(36.0) | 328(31.3) |  |
| Middle | | | 835(26.6) | 270(25.8) | | 268(25.6) | 297(28.4) |  |
| High | | | 1206(38.4) | 381(36.4) | | 403(38.5) | 422(40.3) |  |
| **Cardiovascular risk factors** | | |  |  | |  |  |  |
| Glucose metabolism status | | |  |  | |  |  |  |
| Normal glucose metabolism | | | 1905(60.6) | 526(50.2) | | 685(65.4) | 694(66.3) |  |
| Prediabetes | | | 443(14.1) | 189(18.1) | | 130(12.4) | 124(11.8) |  |
| Type 2 diabetes | | | 772(24.6) | 322(30.8) | | 229(21.9) | 221(21.1) |  |
| Type 1 and other type of diabetes | | | 22(0.7) | 10(1.0) | | 4(0.4) | 8(0.8) |  |
| Mean arterial pressure (mm Hg) | | | 94.82 ± 11.24 | 95.33 ± 11.15 | | 94.53 ± 11.13 | 94.62 ± 11.43 |  |
| Waist circumference (cm) | | | 94.36 ± 13.25 | 95.84 ± 13.45 | | 93.70 ± 13.56 | 93.54 ± 12.59 |  |
| Total/HDL cholesterol ratio | | | 3.51 ± 1.09 | 3.50 ± 1.09 | | 3.50 ± 1.10 | 3.54 ± 1.09 |  |
| Use of lipid-modifying medication (yes vs no) | | | 1007(32.0) | 422(40.3) | | 300(28.6) | 285(27.2) |  |
| **Lifestyle factors** | | |  |  | |  |  |  |
| Alcohol consumption | | |  |  | |  |  |  |
| None | | 524(16.7) | 175(16.7) | | 189(18.0) | 160(15.3) |  |  |
| Low(women<=7,men<=14) | | 1857(59.2) | 602(57.5) | | 596(56.9) | 659(62.9) |  |  |
| High(women>7,men>14) | | 761(24.2) | 270(25.8) | | 263(25.1) | 228(21.8) |  |  |
| Smoking status | | |  |  | |  |  |  |
| Never | | | 1183(37.7) | 361(34.5) | | 397(37.9) | 425(40.6) |  |
| Former | | | 1594(50.7) | 569(54.3) | | 534(51.0) | 491(46.9) |  |
| Current | | | 365(11,6) | 117(11.2) | | 117(11.2) | 131(12.5) |  |
| **Determinant** | | |  |  | |  |  |  |
| Carotid-femoral pulse wave velocity (m/s) | | | 8.98 ± 2.05 | 9.30 ± 2.20 | | 8.86 ± 2.00 | 8.79 ± 1.90 |  |
| **Outcomes** | | |  |  | |  |  |  |
| Retinal thickness indices | | |  |  | |  |  |  |
| mGCL | | 43.95 ± 4.55 | | | 39.21 ± 3.33 | 44.22 ± 1.38 | 48.42 ± 2.57 | |
| mIPL | | 37.49 ± 3.16 | | | 34.23 ± 2.15 | 37.62 ± 1.03 | 40.62 ± 1.94 | |
| mRNFL | | 22.50 ± 4.13 | | | 20.75 ± 1.89 | 22.07 ± 1.55 | 24.30 ± 6.10 | |

Data are presented as mean ± standard deviation, median (interquartile range) or number (%)

Abbreviations: SD, standard deviation; mGCL, macular ganglion cell layer thickness; mIPL, macular innerplexiform layer; mRNFL, macular retinal nerve fiber layer; HDL, high-density lipoprotein.

**Table S2 Additional general study population characteristics based on tertiles of composite Z-score for corneal nerve measures with complete data on carotid femoral pulse wave velocity.**

| **Characteristics** | | **Composite Z-score for corneal nerve measures** | | | | |
| --- | --- | --- | --- | --- | --- | --- |
|  |  | Total study group  (N =3,109) | Tertile 1 (low)  (N = 1036) | Tertile 2 (middle)  (N = 1037) | Tertile 3 (high)  (N = 1036) | |
| **Demographic characteristics** | |  |  |  |  | |
| Age (years) | | 59.94 ± 8.62 | 61.28 ± 8.38 | 59.83 ± 8.57 | 58.72 ± 8.71 | |
| Female, No. (%) | | 1578(50.8) | 444(42.9) | 541(52.2) | 593(57.2) | |
| Educational level | |  |  |  |  | |
| Low | | 1051(33.8) | 358(34.6) | 348(33.6) | 345(33.3) | |
| Middle | | 847(27.2) | 259(25.0) | 288(27.8) | 300(29.0) | |
| High | | 1211(39.0) | 419(40.4) | 401(38.7) | 391(37.7) | |
| **Cardiovascular risk factors** | |  |  |  |  | |
| Glucose metabolism status | |  |  |  |  | |
| Normal glucose metabolism | | 1986(63.9) | 603(58.2) | 651(62.8) | 732(70.7) | |
| Prediabetes | | 452(14.5) | 158(15.3) | 162(15.6) | 132(12.7) | |
| Type 2 diabetes | | 661(21.3) | 269(26.0) | 221(21.3) | 171(16.5) | |
| Type 1 and other type of diabetes | | 10(0.3) | 6(0.6) | 3(0.3) | 1(0.1) | |
| Mean arterial pressure (mm Hg) | | 94.74 ± 11.35 | 95.17 ± 11.43 | 95.22 ± 11.49 | 93.82 ± 11.07 | |
| Waist circumference (cm) | | 94.40 ± 13.03 | 96.01 ± 13.24 | 94.61 ± 13.01 | 92.60 ± 12.61 | |
| Total/HDL cholesterol ratio | | 3.58 ± 1.20 | 3.61 ± 1.11 | 3.57 ± 1.28 | 3.55 ± 1.20 | |
| Use of lipid-modifying medication (yes vs no) | | 893(28.7) | 337(32.5) | 291(28.1) | 265(25.6) | |
| **Lifestyle factors** | |  |  |  |  | |
| Alcohol consumption | |  |  |  |  | |
| None | 551(17.7) | 174(16.8) | 186(17.9) | 191(18.4) | |  |
| Low(women<=7,men<=14) | 1859(59.8) | 624(60.2) | 627(60.5) | 608(58.7) | |  |
| High(women>7,men>14) | 699(22.5) | 238(23.0) | 224(21.6) | 237(22.9) | |  |
| Smoking status | |  |  |  |  | |
| Never | | 1215(39.1) | 371(35.8) | 422(40.7) | 422(40.7) | |
| Former | | 1532(49.3) | 536(51.7) | 501(48.3) | 495(47.8) | |
| Current | | 362(11.6) | 129(12.5) | 114(11.0) | 119(11.5) | |
| **Determinant** | |  |  |  |  | |
| Carotid-femoral pulse wave velocity (m/s) | | 8.89 ± 2.10 | 9.15 ± 2.31 | 8.94 ± 2.14 | 8.59 ± 1.79 | |
| **Outcomes** | |  |  |  |  | |
| Corneal nerve measures | |  |  |  |  | |
| Corneal nerve length | | 13.95 ± 5.10 | 8.36 ± 2.82 | 14.15 ± 1.78 | 19.33 ± 2.62 |  |
| Corneal nerve density | | 75.30 ± 26.21 | 48.72 ± 14.05 | 74.07 ± 10.49 | 103.03 ± 16.64 |  |
| Corneal nerve bifurcation density | | 67.74 ± 41.55 | 27.16 ± 13.72 | 61.67 ± 13.86 | 114.39 ± 30.66 |  |
| Corneal nerve fractal dimension | | 1.31 ± 0.11 | 1.20 ± 0.10 | 1.34 ± 0.05 | 1.42 ± 0.04 |  |
|  | |  |  |  |  | |

Data are presented as mean ± standard deviation, median [interquartile range] or number (%).

Abbreviations: SD, standard deviation; HDL, high-density lipid; m/s, meters per second.

**Table S3 Additional general study population characteristics based on tertiles of composite Z-score for peripheral nerve conduction velocities with complete data on carotid femoral pulse wave velocity.**

| **Characteristic** | | **Composite Z-score for peripheral nerve conduction velocities** | | | | |
| --- | --- | --- | --- | --- | --- | --- |
|  |  | Total study group  (N =3722) | Tertile 1 (low)  (N = 1240) | Tertile2 (middle)  (N = 1241) | Tertile 3 (high)  (N = 1241) | |
| **Demographic characteristics** | |  |  |  |  | |
| Age (years) | | 59.27 ± 8.61 | 61.78 ± 7.78 | 59.16 ± 8.66 | 56.89 ± 8.65 | |
| Female, No. (%) | | 1932(51.9) | 349(28.1) | 662(53.3) | 921(74.2) | |
| Educational level | |  |  |  |  | |
| Low | | 1201(32.2) | 421(34.0) | 388(31.3) | 392(31.6) | |
| Middle | | 1029(27.7) | 321(25.9) | 329(26.5) | 379(30.5) | |
| High | | 1492(40.1) | 498(40.2) | 524(42.2) | 470(37.9) | |
| **Cardiovascular risk factors** | |  |  |  |  | |
| Glucose metabolism status | |  |  |  |  | |
| Normal glucose metabolism | | 2436(65.4) | 659(53.1) | 842(67.8) | 939(75.9) | |
| Prediabetes | | 539(14.5) | 188(15.2) | 180(14.5) | 169(13.7) | |
| Type 2 diabetes | | 730(19.5) | 381(30.7) | 214(17.2) | 129(10.4) | |
| Type 1 and other type of diabetes | | 17(0.5) | 12(1.0) | 5(0.4) | - | |
| Mean arterial pressure (mm Hg) | | 94.53 ± 11.33 | 95.88 ± 11.04 | 94.59 ± 11.15 | 93.11 ± 11.61 | |
| Waist circumference (cm) | | 93.25 ± 12.58 | 97.19 ± 12.36 | 92.92 ± 12.14 | 89.64 ± 12.10 | |
| Total/HDL cholesterol ratio | | 3.58 ± 1.21 | 3.63 ± 1.24 | 3.56 ± 1.22 | 3.55 ± 1.17 | |
| Use of lipid-modifying medication (yes vs no) | | 1038(27.9) | 460(37.1) | 335(27.0) | 243(19.6) | |
| **Lifestyle factors** | |  |  |  |  | |
| Alcohol consumption | |  |  |  |  | |
| None | 643(17.3) | 185(14.9) | 217(17.5) | 241(19.4) | |  |
| Low(women <=7,men <=14) | 2215(59.5) | 736(59.4) | 747(60.2) | 732(59.0) | |  |
| High(women >7,men >14) | 864(23.2) | 319(25.7) | 277(22.2) | 268(21.6) | |  |
| Smoking status | |  |  |  |  | |
| Never | | 1444(38.8) | 423(34.1) | 488(39.3) | 533(42.9) | |
| Former | | 1815(48.8) | 660(53.2) | 583(47.0) | 572(46.1) | |
| Current | | 463(12.4) | 157(12.7) | 170(13.7) | 136(11.0) | |
| **Determinant** | |  |  |  |  | |
| Carotid-femoral pulse wave velocity (m/s) | | 8.82 ± 2.01 | 9.34 ± 2.19 | 8.80 ± 2.04 | 8.33 ± 1.62 | |
| **Outcomes** | |  |  |  |  | |
| Peripheral nerve conduction velocities | |  |  |  |  | |
| Peroneal nerve conduction velocity (m/s) | | 46.57 ± 4.39 | 42.58 ± 3.48 | 46.77 ± 2.43 | 50.37 ± 3.06 |  |
| Tibial nerve conduction velocity (m/s) | | 44.83 ± 4.48 | 40.98 ± 3.26 | 44.81 ± 2.53 | 48.70 ± 3.65 |  |
| Sural nerve conduction velocity (m/s) | | 44.30 ± 6.04 | 43.54 ± 4.11 | 48.29 ± 3.75 | 53.08 ± 5.74 |  |
| Peripheral nerve amplitudes | |  |  |  |  |  |
| Peroneal amplitude (mV) | | 5.03 ± 2.01 | 4.59 ± 2.00 | 5.10 ± 1.94 | 5.38 ± 2.02 |  |
| Tibial amplitude (mV) | | 10.07 ± 4.05 | 8.55 ± 3.84 | 10.35 ± 3.91 | 11.30 ± 3.92 |  |
| Sural amplitude (mV) | | 9.69 ± 6.41 | 8.39 ± 5.45 | 9.65 ± 6.23 | 11.03 ± 7.17 |  |

Data are presented as mean ± standard deviation, median [interquartile range] or number (%).

Abbreviations: SD, standard deviation; HDL, high-density lipid; m/s, meters per second; mV, millivolts.

**Table S4 Additional general study population characteristics based on tertiles of mean peripheral vibration perception thresholds in the study population with complete data on carotid-femoral pulse wave velocity.**

| **Characteristic** | | **Mean peripheral vibration perception thresholds** | | | |
| --- | --- | --- | --- | --- | --- |
|  |  | Total study group  (N =5140) | Tertile 1 (low)  (N = 1719) | Tertile2 (middle)  (N = 1714) | Tertile 3 (high)  (N = 1708) |
| **Demographic characteristics** | |  |  |  |  |
| Age (years) | | 60.10 ± 8.59 | 55.31 ± 8.47 | 60.47 ± 7.65 | 64.56 ± 6.96 |
| Female, No. (%) | | 2598(50.5) | 1106(64.4) | 919(53.6) | 573(33.5) |
| Educational level | |  |  |  |  |
| Low | | 1758(34.2) | 478(27.8) | 614(35.8) | 666(39.0) |
| Middle | | 1414(27.5) | 527(30.7) | 455(26.5) | 432(25.3) |
| High | | 1968(38.3) | 713(41.5) | 645(37.6) | 610(35.7) |
| **Cardiovascular risk factors** | |  |  |  |  |
| Glucose metabolism status | |  |  |  |  |
| Normal glucose metabolism | | 3179(61.8) | 1260(73.3) | 1058(61.7) | 861(50.4) |
| Prediabetes | | 748(14.6) | 209(12.2) | 258(15.1) | 281(16.5) |
| Type 2 diabetes | | 1184(23.0) | 241(14.0) | 389(22.7) | 554(32.4) |
| Type 1 and other type of diabetes | | 29(0.6) | 8(0.5) | 9(0.5) | 12(0.7) |
| Mean arterial pressure (mm Hg) | | 94.78 ± 11.32 | 93.35 ± 11.23 | 95.26 ± 11.52 | 95.75 ± 11.08 |
| Waist circumference (cm) | | 94.67 ± 13.35 | 90.47 ± 12.11 | 93.90 ± 12.88 | 99.68 ± 13.39 |
| Total/HDL cholesterol ratio | | 3.57 ± 1.17 | 3.54 ± 1.15 | 3.56 ± 1.19 | 3.61 ± 1.16 |
| Use of lipid-modifying medication (yes vs no) | | 1568(30.5) | 335(19.5) | 536(31.3) | 697(40.8) |
| **Lifestyle factors** | |  |  |  |  |
| Alcohol consumption | |  |  |  |  |
| None | 915(17.8) | 322(18.7) | 313(18.3) | 280(16.4) |  |
| Low(women<=7,men<=14) | 3042(59.2) | 1025(59.7) | 1002(58.5) | 1015(59.4) |  |
| High(women>7,men>14) | 1183 (23.0) | 371(21.6) | 399(23.3) | 413(24.2) |  |
| Smoking status | |  |  |  |  |
| Never | | 1954(38.0) | 745(43.4) | 646(37.7) | 563(33.0) |
| Former | | 2561(49.8) | 759(44.2) | 862(50.3) | 940(55.0) |
| Current | | 625(12.2) | 214(12.5) | 206(12.0) | 205(12.0) |
| **Determinant** | |  |  |  |  |
| Carotid-femoral pulse wave velocity (m/s) | | 8.95 ± 2.11 | 8.34 ± 1.75 | 8.98 ± 2.13 | 9.55 ± 2.25 |
| **Outcomes** | |  |  |  |  |
| Vibration perception threshold | |  |  |  |  |
| NTM left | | 12.94 ± 8.32 | 5.95 ± 1.69 | 10.78 ± 2.61 | 22.13 ± 7.78 |
| NTM right | | 12.89 ± 8.32 | 5.99 ± 1.68 | 10.76 ± 2.65 | 21.99 ± 7.97 |
|  | |  |  |  |  |

Data are presented as mean ± standard deviation, median [interquartile range] or number (%).

Abbreviations: SD, standard deviation; NTM, neurothesiometer; m/s, meters per second; HDL, high-density lipid.

**Table S5 Associations of carotid-femoral pulse wave velocity with both individual components and composite z-scores for retinal nerve layer thickness, retinal sensitivity, corneal nerve measures, peripheral nerve conduction velocities, amplitudes and peripheral vibration perception thresholds.**

| **Individual components of retinal nerve layer thickness (N=3,142)** | | | | | | | | | | | | |
| --- | --- | --- | --- | --- | --- | --- | --- | --- | --- | --- | --- | --- |
|  |  |  | Composite Z-score for retinal nerve layer thickness | | Macular retinal nerve fiber layer thickness | | Macular ganglion cell layer thickness | | Macular inner plexiform layer thickness | |  |  |
|  |  | Model | stβ (95%CI) | P-value | stβ (95%CI) | P-value | stβ (95%CI) | P-value |  |  |  |  |
| cfPWV, per SD | | 1 | **-0.09 (-0.12 to -0.06)** | **<0.001** | -0.01 (-0.04 to 0.03) | 0.750 | **-0.13 (-0.17 to -0.09)** | **<0.001** | **-0.14 (-0.17 to -0.10)** | **<0.001** |  |  |
|  |  | 2 | **-0.04 (-0.07 to -0.01)** | **0.014** | **-0.04 (-0.08 to -0.002)** | **0.040** | -0.04 (-0.08 to 0.01) | 0.089 | **-0.05 (-0.09 to -0.01)** | **0.017** |  |  |
|  |  | 3 | **-0.04 (-0.07 to -0.004)** | **0.031** | **-0.04 (-0.08 to -0.001)** | **0.048** | -0.03 (-0.07 to 0.01) | 0.167 | **-0.04 (-0.08 to -0.002)** | **0.042** |  |  |
| **Individual components of retinal sensitivity (N=4531)** | | | | | | | | | | | | |
|  |  |  | Composite Z-score for retinal sensitivity of both eyes | | Retinal sensitivity of right eye | | Retinal sensitivity of left eye | |  |  |  |  |
|  |  | Model | stβ (95%CI) | P-value | stβ (95%CI) | P-value | stβ (95%CI) | P-value |  |  |  |  |
| cfPWV, per SD |  | 1 | **-0.19 (-0.22 to -0.17)** | **<0.001** | **-0.19 (-0.22 to -0.17)** | **<0.001** | **-0.16 (-0.19 to -0.13)** | **<0.001** |  |  |  |  |
|  |  | 2 | **-0.07 (-0.09 to -0.03)** | **<0.001** | **-0.07 (-0.10 to -0.04)** | **<0.001** | **-0.05 (-0.08 to -0.01)** | **0.007** |  |  |  |  |
|  |  | 3 | **-0.06 (-0.09 to -0.03)** | **<0.001** | **-0.07 (-0.10 to -0.04)** | **<0.001** | **-0.04 (-0.08 to -0.01)** | **0.012** |  |  |  |  |
| **Individual components of corneal nerve measures (N=3,109)** | | | | | | | | | | | | |
|  |  |  | Composite Z-score for corneal nerve measures | | Corneal nerve  length | | Corneal nerve  density | | Corneal nerve  birfucation density | | Corneal nerve fractal dimension | |
|  |  | Model | stβ (95%CI) | P-value | stβ (95%CI) | P-value | stβ (95%CI) | P-value | stβ (95%CI) | P-value | stβ (95%CI) | P-value |
| cfPWV, per SD | | 1 | **-0.12 (-0.15 to -0.08)** | **<0.001** | **-0.13 (-0.16 to -0.09)** | **<0.001** | **-0.11 (-0.14 to -0.07)** | **<0.001** | **-0.09 (-0.13 to -0.06)** | **<0.001** | **-0.14 (-0.17 to -0.10)** | **<0.001** |
|  |  | 2 | **-0.06 (-0.09 to -0.02)** | **0.005** | **-0.05 (-0.09 to -0.01)** | **0.015** | **-0.05 (-0.09 to -0.01)** | **0.022** | **-0.04 (-0.08 to -0.0004)** | **0.048** | **-0.08 (-0.12 to -0.04)** | **<0.001** |
|  |  | 3 | **-0.05 (-0.09 to -0.01)** | **0.009** | **-0.05 (-0.09 to -0.004)** | **0.029** | **-0.04 (-0.08 to -0.002)** | **0.039** | -0.04 (-0.08 to 0.003) | 0.073 | **-0.08 (-0.12 to -0.04)** | **<0.001** |
| **Individual components of peripheral nerve conduction velocities (N=3,722)** | | | | | | | | | | | | |
|  |  |  | Composite Z-score for peripheral NCV | | Peroneal NCV | | Tibial NCV | | Sural NCV | |  |  |
|  |  | Model | stβ (95%CI) | P-value | stβ (95%CI) | P-value | stβ (95%CI) | P-value | stβ (95%CI) | P-value |  |  |
| cfPWV, per SD | | 1 | **-0.16 (-0.19 to -0.14)** | **<0.001** | **-0.19 (-0.22 to -0.16)** | **<0.001** | **-0.15 (-0.18 to -0.12)** | **<0.001** | **-0.15 (-0.18 to -0.11)** | **<0.001** |  |  |
|  |  | 2 | **-0.05 (-0.08 to -0.02)** | **<0.001** | **-0.06 (-0.09 to -0.02)** | **0.001** | **-0.04 (-0.07 to -0.001)** | **0.045** | **-0.05 (-0.09 to -0.02)** | **0.004** |  |  |
|  |  | 3 | **-0.05 (-0.08 to -0.02)** | **<0.001** | **-0.06 (-0.09 to -0.02)** | **0.001** | **-0.04 (-0.08 to -0.01)** | **0.023** | **-0.06 (-0.09 to -0.02)** | **0.003** |  |  |
| **Peripheral nerve amplitudes (N-3,722)** | | | | | | | | | | | | |
|  |  |  | Peroneal CMAP amplitude | | Tibial CMAP amplitude | | Sural SNAP amplitude | |  |  |  |  |
|  |  | Model | stβ (95%CI) | P-value | stβ (95%CI) | P-value | stβ (95%CI) | P-value |  |  |  |  |
| cfPWV, per SD | | 1 | **-0.08 (-0.11 to -0.05)** | **<0.001** | **-0.19 (-0.23 to -0.16)** | **<0.001** | **-0.13 (-0.16 to -0.09)** | **<0.001** |  |  |  |  |
|  |  | 2 | -0.01 (-0.04 to 0.03) | 0.714 | **-0.04 (-0.08 to -0.01)** | **0.023** | 0.00 (-0.04 to 0.03) | 0.841 |  |  |  |  |
|  |  | 3 | 0.00 (-0.04 to 0.04) | 1,000 | **-0.05 (-0.08 to -0.01)** | **0.009** | 0.00 (-0.04 to 0.03) | 0.896 |  |  |  |  |
| **Individual components of peripheral vibration perception thresholds (N=5,140)** | | | | | | | | | | | | |
|  |  |  | Composite Z-score vibration perception threshold of right and left first toe | | Vibration perception threshold of right first toe | | Vibration perception threshold of left first toe | |  |  |  |  |
|  |  | Model | Beta (95%CI) | P-value | Beta (95%CI) | P-value | Beta (95%CI) | P-value |  |  |  |  |
| cfPWV, per SD | | 1 | **0.27 (0.24 to 0.29)** | **<0.001** | **0.25 (0.22 to 0.28)** | **<0.001** | **0.26 (0.24 to 0.29)** | **<0.001** |  |  |  |  |
|  |  | 2 | **0.06 (0.03 to 0.09)** | **<0.001** | **0.06 (0.03 to 0.09)** | **<0.001** | **0.06 (0.03 to 0.09)** | **<0.001** |  |  |  |  |
|  |  | 3 | **0.06 (0.03 to 0.08)** | **<0.001** | **0.05 (0.02 to 0.08)** | **<0.001** | **0.05 (0.03 to 0.08)** | **<0.001** |  |  |  |  |

1 SD corresponds with 0.8 (unit-less) for composite Z-score for retinal nerve layer thickness measures 4.1 micrometer macular retinal nerve fiber layer thickness, 4.6 micrometer macular ganglion cell layer thickness, 16.7 micrometer total retinal layer thickness, 3.2 micrometer macular inner plexiform layer thickness, 2.0 dB for retinal sensitivity, 0.9 (unit-less) for composite Z-score for corneal nerve measures, 41.5 number of branches/mm^2^ for corneal nerve bifurcation density, 26.2 number of main fibers/mm^2^ for corneal nerve density, 0.1 (unit-less) for corneal nerve fractal dimension, 5.1 mm/mm^2^ for corneal nerve length, 0.8 (unit-less) for composite Z-score for peripheral nerve conduction velocity measures, 4.4 m/s for peroneal nerve conduction velocity, 4.5 m/s for tibial nerve conduction velocity, 6.0 m/s for sural nerve conduction velocity, 2.0 mV for peroneal nerve CMAP amplitude , 4.0 mV for tibial nerve CMAP amplitude, 6.4 µV for sural nerve SNAP amplitude, 8.0 volts for vibration perception threshold and 2.1 m/s for carotid-femoral pulse wave velocity.

Variables entered in models: Model 1: crude; Model 2: adjusted for age, sex, glucose metabolism status, educational level, mean arterial pressure; Model 3: additionally adjusted for waist circumference, alcohol consumption status, smoking status, total cholesterol-to-HDL cholesterol ratio, use of lipid-modifying medication, Dutch Health Diet score, and accelerometer-assessed physical activity.

Bold denotes P<0.05.

Abbreviations: stβ, standardized beta: CI, confidence interval; SD, standard deviation; N, population sample size; HDL, high-density lipoprotein; dB, decibel; m/s, meters per second; NCV, nerve conduction velocity; m/s, meters per second; CMAP, compound muscle action potential; SNAP, sensory nerve action potential; m/s, meters per second; mV, millivolts; µV, microvolts.

**Table S6 Associations of carotid-femoral pulse wave velocity with composite z-scores for retinal nerve layer thickness, retinal sensitivity, corneal nerve measures, peripheral nerve conduction velocities and peripheral vibration perception threshold additionally adjusted for history of cardiovascular disease, chronic kidney disease, use of diabetes medication (model 4A); use of specific anti-hypertensive medications (ACE inhibitors, angiotensin receptor blockers (ARBs), aldosterone antagonists (model 4B); and the use of other groups of anti-hypertensive medications (model 4C).**

|  | | | | | | | | | | | | |
| --- | --- | --- | --- | --- | --- | --- | --- | --- | --- | --- | --- | --- |
|  |  |  | Composite Z-score for retinal nerve layer thickness  N=3,130 | | Composite Z-score for retinal sensitivity of both eyes  N=4,515 | | Composite Z-score for corneal nerve measures  N=3,096 | | Composite Z-score for peripheral NCV  N=3,709 | | Composite Z-score vibration perception threshold of right and left first toe  N=5,116 |  |
|  |  | Model | stβ (95%CI) | P-value | stβ (95%CI) | P-value | stβ (95%CI) | P-value | stβ (95%CI) |  | stβ (95%CI) | P-value |
| cfPWV, per SD | | 4a | **-0.04 (-0.07 to -0.001)** | **0.044** | **-0.06 (-0.09 to -0.03)** | **<0.001** | **-0.05 (-0.09 to -0.01)** | **0.013** | **-0.05 (-0.08 to -0.02)** | **<0.001** | **0.05 (0.02 to 0.08)** | **<0.001** |
|  |  | 4b | **-0.04 (-0.07 to -0.001)** | **0.045** | **-0.06 (-0.09 to -0.03)** | **<0.001** | **-0.05 (-0.09 to -0.01)** | **0.011** | **-0.05 (-0.07 to -0.02)** | **<0.001** | **0.05 (0.02 to 0.07)** | **<0.001** |
|  |  | 4c | **-0.04 (-0.07 to -0.002)** | **0.040** | **-0.06 (-0.09 to -0.03)** | **<0.001** | **-0.05 (-0.09 to -0.01)** | **0.011** | **-0.05 (-0.07 to -0.02)** | **<0.001** | **0.05 (0.02 to 0.08)** | **<0.001** |
|  |  |  |  |  |  |  |  |  |  |  |  |  |

1 SD corresponds with 0.8 (unit-less) for composite Z-score for retinal nerve layer thickness measures, 2.0 dB for retinal sensitivity, 0.9 (unit-less) for composite Z-score for corneal nerve measures, 0.8 (unit-less) for composite Z-score for peripheral nerve conduction velocity measures, 8.0 volts for vibration perception threshold and 2.1 m/s for carotid-femoral pulse wave velocity.

Variables entered in models: Model 4a: crude, adjusted for age, sex, glucose metabolism status, educational level, mean arterial pressure, waist circumference, alcohol consumption status, smoking status, total cholesterol-to-HDL cholesterol ratio, use of lipid-modifying medication, Dutch Health Diet score, accelerometer-assessed physical activity, history of cardiovascular disease, chronic kidney disease [yes/no] and use of diabetes medication;

Model 4b: additionally adjusted for use of specific anti-hypertensive medications [ACE inhibitors, Angiotensin receptor blockers (ARBs), and Aldosterone antagonists];

Model 4c: additionally adjusted for use of other groups of anti-hypertensive medications.

Bold denotes P<0.05.

Abbreviations: stβ, standardized beta: CI, confidence interval; SD, standard deviation; N, population sample size; HDL, high-density lipoprotein; ACE, angiotensin converting enzyme; dB, decibel; m/s, meters per second; NCV, nerve conduction velocity; m/s, meters per second; CMAP, compound muscle action potential; SNAP, sensory nerve action potential; m/s, meters per second; mV, millivolts; µV, microvolts.

**Table S7 Associations of carotid-femoral pulse wave velocity with peripheral nerve amplitudes, additionally adjusted for history of cardiovascular disease, chronic kidney disease, use of diabetes medication (model 4A); use of specific anti-hypertensive medications (ACE inhibitors, angiotensin receptor blockers (ARBs), aldosterone antagonists (model 4B); and the use of other groups of anti-hypertensive medications (model 4C); where waist circumference was replaced with BMI (model 5A), or glucose metabolism status was replaced with fasting plasma glucose (model 5B), with HbA1c (model 5C), or educational level was replaced with income level (model 5D) and office mean arterial pressure was replaced by 24-hour ambulatory mean arterial pressure (model 5E).**

| N= |  | Peroneal CMAP amplitude | | Tibial CMAP amplitude | | Sural SNAP amplitude | |
| --- | --- | --- | --- | --- | --- | --- | --- |
|  | Model | stβ (95%CI) | P-value | stβ (95%CI | P-value | stβ (95%CI | P-value |
| cfPWV, per SD | 4a | 0.004 (-0.03 to 0.04) | 0.849 | **-0.05 (-0.08 to -0.01)** | 0.007 | -0.003 (-0.04 to 0.03) | 0.858 |
|  | 4b | 0.005 (-0.03 to 0.04) | 0.809 | **-0.05 (-0.09 to -0.01)** | 0.006 | -0.004 (-0.04 to 0.03) | 0.834 |
|  | 4c | 0.005 (-0.03 to 0.04) | 0.813 | **-0.05 (-0.09 to -0.01)** | 0.006 | -0.004 (-0.04 to 0.03) | 0.830 |
|  | | | | | | | |
|  |  | Peroneal CMAP amplitude | | Tibial CMAP amplitude | | Sural SNAP amplitude | |
| cfPWV, per SD | †Model | stβ (95%CI) | N | stβ (95%CI) | N | stβ (95%CI) | N |
|  | 5a | 0.001 (-0.04; 0.04) | 3,721 | **-0.05 (-0.09; -0.02)** | 3,721 | -0.004 (-0.04; 0.03) | 3,721 |
|  | 5b | 0.002 (-0.04; 0.04) | 3,718 | **-0.05 (-0.09; -0.02)** | 3,718 | -0.004 (-0.04; 0.03) | 3,718 |
|  | 5c | 0.002 (-0.04; 0.04) | 3,718 | **-0.05 (-0.09; -0.02)** | 3,718 | -0.004 (-0.04; 0.03) | 3,718 |
|  | 5d | -0.01 (-0.05; 0.03) | 2,899 | **-0.06 (-0.10; -0.02)** | 2,899 | 0.01 (-0.04; 0.05) | 2,899 |
|  | 5e | 0.001 (-0.04; 0.04) | 3,373 | **-0.05 (-0.08; -0.01)** | 3,373 | 0.003 (-0.04; 0.04) | 3,373 |

Table S7 shows the associations of carotid-femoral pulse wave velocity with peripheral nerve amplitudes. Standardized regression coefficients (stβ) represent the differences in peripheral nerve conduction velocity measures in SD for carotid-femoral pulse wave velocity. 1 SD corresponds with 2.0 mV for peroneal nerve CMAP amplitude , 4.0 mV for tibial nerve CMAP amplitude, 6.4 µV for sural nerve SNAP amplitude, 2.0 m/s for carotid-femoral pulse wave velocity.

Variables entered in models: Model 4a: crude, adjusted for age, sex, glucose metabolism status, educational level, mean arterial pressure, waist circumference, alcohol consumption status, smoking status, total cholesterol-to-HDL cholesterol ratio, use of lipid-modifying medication, Dutch Health Diet score, accelerometer-assessed physical activity, history of cardiovascular disease, chronic kidney disease [yes/no] and use of diabetes medication;

Model 4b: additionally adjusted for use of specific anti-hypertensive medications [ACE inhibitors, Angiotensin receptor blockers (ARBs), and Aldosterone antagonists];

Model 4c: additionally adjusted for use of other groups of anti-hypertensive medications.

† Variables entered in model 5: crude + adjusted for age, sex, (fasting plasma glucose or HbA1c), income level, 24-hour mean arterial pressure; BMI, alcohol consumption status, smoking status, total cholesterol-to-HDL cholesterol ratio, use of lipid-modifying medication, Dutch Health Diet score, and accelerometer-assessed physical activity.

Bold denotes P<0.05.

Abbreviations: stβ, standardized beta: CI, confidence interval; SD, standard deviation; N, population sample size; HDL, high-density lipoprotein; CMAP, compound muscle action potential; SNAP, sensory nerve action potential; m/s, meters per second; mV, millivolts; µV, microvolts.

**Table S8 Associations of carotid-femoral pulse wave velocity with composite Z-score for retinal nerve layer thickness, retinal sensitivity, corneal nerve measures, peripheral nerve conduction velocities and vibration perception threshold where waist circumference was replaced with BMI (model 5A), or glucose metabolism status was replaced with fasting plasma glucose (model 5B), with HbA1c (model 5C), or educational level was replaced with income level (model 5D) and office mean arterial pressure was replaced by 24-hour ambulatory mean arterial pressure (model 5E), with corneal nerve measures additionally adjusted for CCM lag time(model 6), peripheral nerve conduction velocities additionally adjusted for height(model 7), and finally all composite measures additionally adjusted for heart rate(model 8).**

| **Associations of carotid-femoral pulse wave velocity with composite Z-score for retinal nerve layer thickness, retinal sensitivity, corneal nerve measures, peripheral nerve conduction velocities and vibration perception threshold where waist circumference was replaced with BMI (model 5A), or glucose metabolism status was replaced with fasting plasma glucose (model 5B), with HbA1c (model 5C), or educational level was replaced with income level (model 5D) and office mean arterial pressure was replaced by 24-hour ambulatory mean arterial pressure (model 5E)** | | | | | | | | | | | | |
| --- | --- | --- | --- | --- | --- | --- | --- | --- | --- | --- | --- | --- |
|  |  | Composite Z-score for retinal nerve layer thickness | | Composite Z-score for retinal sensitivity of both eyes | | Composite Z-score for corneal nerve measures | | | Composite Z-score for peripheral NCV | | Composite Z-score vibration perception threshold of right and left first toe | |
|  | †Model | stβ (95%CI) | N | stβ (95%CI) | N | stβ (95%CI) | | N | stβ (95%CI) | N | stβ (95%CI) | N |
| cfPWV, per SD | 5a | **-0.04 (-0.07; -0.003)** | 3,130 | **-0.06 (-0.09; -0.03)** | 4,518 | **-0.05 (-0.09; -0.01)** | | 3,099 | **-0.05 (-0.07; -0.02)** | 3,709 | **0.05 (0.02; 0.08)** | 5,118 |
|  | 5b | **-0.04 (-0.07; -0.01)** | 3,130 | **-0.06 (-0.09; -0.03)** | 4,516 | **-0.05 (-0.09; -0.01)** | | 3,097 | **-0.05 (-0.08; -0.02)** | 3,707 | **0.05 (0.02; 0.07)** | 5,116 |
|  | 5c | **-0.04 (-0.07; -0.01)** | 3,129 | **-0.06 (-0.09; -0.03)** | 4,515 | **-0.05 (-0.09; -0.01)** | | 3,096 | **-0.05 (-0.07; -0.02)** | 3,707 | **0.05 (0.02; 0.07)** | 5,115 |
|  | 5d | -0.03 (-0.07; 0.01) | 2,436 | -0.04 (-0.08; 0.001) | 3,491 | **-0.06 (-0.10; -0.02)** | | 2,419 | **-0.05 (-0.08; -0.02)** | 2,902 | **0.04 (0.01; 0.07)** | 3,930 |
|  | 5e | **-0.05 (-0.09; -0.01)** | 2,832 | **-0.05 (-0.08; -0.01)** | 4,079 | **-0.04 (-0.08; -0.002)** | | 2,840 | **-0.04 (-0.07; -0.02)** | 3,364 | **0.03 (0.01; 0.05)** | 4,615 |
|  | | | | | | | | | | | | |
| **Associations of carotid-femoral pulse wave velocity with corneal nerve measures additionally adjusted for CCM lag time.** | | | | | | | | | | | | |
|  |  | Composite Z-score for corneal nerve measures | |  |  |  | |  |  |  |  |  |
|  | ‡ Model 6 | stβ (95%CI) | N |  |  |  | |  |  |  |  |  |
| cfPWV, per SD |  | **-0.09 (-0.16; -0.01)** | 795 |  |  |  | |  |  |  |  |  |
|  | | | | | | | | | | | | |
|  | | | | | | | | | | | | |
| **Associations of carotid-femoral pulse wave velocity with peripheral nerve conduction velocities additionally adjusted for height** | | | | | | | | | | | | |
|  |  | Composite Z-score for peripheral NCV | |  |  |  | |  |  |  |  |  |
|  | § Model 7 | stβ (95%CI) | N |  |  |  | |  |  |  |  |  |
| cfPWV, per SD |  | **-0.03 (-0.06; -0.01)** | 3,708 |  |  |  | |  |  |  |  |  |
|  | | | | | | | | | | | | |
| **Associations of carotid-femoral pulse wave velocity with Retinal nerve layer thickness, retinal sensitivity, corneal nerve measures, peripheral nerve conduction velocities and peripheral vibration perception threshold additionally adjusted for heart rate** | | | | | | | | | | | | |
|  |  | Composite Z-score for retinal nerve layer thickness | | Composite Z-score for retinal sensitivity of both eyes | | Composite Z-score for corneal nerve measures | | | Composite Z-score for peripheral NCV | | Composite Z-score vibration perception threshold of right and left first toe | |
|  | # Model | stβ (95%CI) | P-value | stβ (95%CI) | P-value | stβ (95%CI) | P-value | | stβ (95%CI) | P-value | stβ (95%CI) | P-value |
| cfPWV, per SD | 1 | **-0.10 (-0.13 to -0.07)** | <0.001 | **-0.19 (-0.22 to -0.16)** | <0.001 | **-0.12 (-0.15 to -0.09)** | | <0.001 | **-0.16 (-0.19 to -0.14)** | <0.001 | **0.27 (0.24 to 0.29)** | <0.001 |
|  | 2 | **-0.04 (-0.08 to -0.01)** | 0.023 | **-0.07 (-0.10 to -0.03)** | <0.001 | **-0.05 (-0.09 to -0.01)** | | 0.019 | **-0.16 (-0.19 to -0.14)** | <0.001 | **0.06 (0.03 to 0.09)** | <0.001 |
|  | 3 | **-0.04 (-0.07 to -0.002)** | 0.040 | **-0.06 (-0.09 to -0.03)** | <0.001 | **-0.05 (-0.09 to -0.01)** | | 0.028 | **-0.16 (-0.19 to -0.14)** | <0.001 | **0.06 (0.03 to 0.09)** | <0.001 |
|  | | | | | | | | | | | | |
| **Associations of carotid-femoral pulse wave velocity with composite z-score for both retinal nerve layer thickness and corneal nerve measures additionally adjusted for intraocular pressure** | | | | | | | | | | | | |
|  |  | Composite Z-score for retinal nerve layer thickness | | Composite Z-score for corneal nerve measures | |  | |  |  |  |  |  |
|  | \|\|Model | stβ (95%CI) | P-value | stβ (95%CI) | P-value |  | |  |  |  |  |  |
| cfPWV, per SD | 1 | **-0.11 (-0.14 to -0.08)** | **<0.001** | **-0.12 (-0.15 to -0.09)** | **<0.001** |  | |  |  |  |  |  |
|  | 2 | **-0.06 (-0.10 to -0.02)** | **0.002** | **-0.05 (-0.09 to -0.01)** | **0.008** |  | |  |  |  |  |  |
|  | 3 | **-0.05 (-0.09 to -0.02)** | **0.006** | **-0.05 (-0.09 to -0.01)** | **0.024** |  | |  |  |  |  |  |

1 SD corresponds with 0.8 (unit-less) for composite Z-score for retinal nerve layer thickness measures, 2.0 dB for retinal sensitivity, 0.9 (unit-less) for composite Z-score for corneal nerve measures, 0.8 (unit-less) for composite Z-score for peripheral nerve conduction velocity measures, 8.0 volts for vibration perception threshold, and 2.1 m/s for carotid-femoral pulse wave velocity.

In Models 5A, 5B, 5C, 5D and 5E values per SD were numerically comparable.

Bold denotes P<0.05.

† Variables entered in model: crude + adjusted for age, sex, glucose metabolism status, educational level, mean arterial pressure, waist circumference, alcohol consumption status, smoking status, total cholesterol-to-HDL cholesterol ratio, use of lipid-modifying medication, Dutch Health Diet score and accelerometer-assessed physical activity.

‡ Variables entered in model 6: crude + adjusted for age, sex, glucose metabolism status, CCM lag time, educational level, mean arterial pressure, waist circumference, alcohol consumption status, smoking status, total cholesterol-to-HDL cholesterol ratio, use of lipid-modifying medication, Dutch Health Diet score and accelerometer-assessed physical activity.

§ Variables entered in model 7: crude + adjusted for age, sex, glucose metabolism status, educational level, mean arterial pressure, waist circumference, alcohol consumption status, smoking status, total cholesterol-to-HDL cholesterol ratio, use of lipid-modifying medication, Dutch Health Diet score and accelerometer-assessed physical activity.

# Variables entered in models: Model 1: crude; Model 2: adjusted for age, sex, glucose metabolism status, educational level, mean arterial pressure, heart rate; Model 3: additionally adjusted for waist circumference, alcohol consumption status, smoking status, total cholesterol-to-HDL cholesterol ratio, use of lipid-modifying medication, Dutch Health Diet score, and accelerometer-assessed physical activity.

|| Variables entered in models: Model 1: crude; Model 2: adjusted for age, sex, glucose metabolism status, educational level, mean arterial pressure, heart rate; Model 3: additionally adjusted for waist circumference, alcohol consumption status, smoking status, total cholesterol-to-HDL cholesterol ratio, use of lipid-modifying medication, Dutch Health Diet score, accelerometer-assessed physical activity and intraocular pressure.

Abbreviations: stβ, standardized beta; CI, confidence interval; SD, standard deviation; HDL, high-density lipoprotein; N, population size; decibel; m/s, meters per second; NCV, nerve conduction velocity; m/s, meters per second; CMAP, compound muscle action potential; SNAP, sensory nerve action potential; m/s, meters per second; mV, millivolts; µV, microvolts; CCM, corneal confocal microscopy; NCV, nerve conduction velocity.

**Table S9 Associations of carotid-femoral pulse wave velocity with retinal sensitivity excluding images with percentage false positives >15% and percentage false negatives >30%.**

|  | | | | | | | | | |
| --- | --- | --- | --- | --- | --- | --- | --- | --- | --- |
|  |  |  | Composite Z-score for retinal nerve layer thickness | | Retinal sensitivity of right eye | | Retinal sensitivity of left eye | |  |
|  |  | Model | stβ (95%CI) | P-value | stβ (95%CI) | P-value | stβ (95%CI) | P-value |  |
| cfPWV, per SD | | 1 | **-0.19 (-0.23 to -0.17)** | <0.001 | **-0.19 (-0.23 to -0.17)** | <0.001 | **-0.16 (-0.19 to -0.13)** | <0.001 |  |
|  |  | 2 | **-0.04 (-0.07 to -0.01)** | 0.015 | **-0.05 (-0.08 to -0.02)** | 0.004 | -0.02 (-0.06 to 0.01) | 0.172 |  |
|  |  | 3 | **-0.04 (-0.07 to -0.004)** | 0.026 | **-0.05 (-0.08 to -0.011)** | 0.009 | -0.02 (-0.06 to 0.01) | 0.222 |  |

Standardized regression coefficients (stβ) represent the differences in retinal sensitivity in SD for carotid-femoral pulse wave velocity. 1 SD corresponds with 1.4 dB for mean retinal sensitivity of both eyes, 1.6 dB for mean retinal sensitivity of right eye, 1.5 dB for mean retinal sensitivity of left eye, 2.0 m/s for carotid-femoral pulse wave velocity.

Variables entered in models: Model 1: crude; Model 2: adjusted for age, sex, glucose metabolism status, educational level, mean arterial pressure; Model 3: additionally adjusted for waist circumference, alcohol consumption status, smoking status, total cholesterol-to-HDL cholesterol ratio, use of lipid-modifying medication, Dutch Health Diet score, and accelerometer-assessed physical activity.

Bold denotes P<0.05.

Abbreviations: stβ, standardized beta: CI, confidence interval; SD, standard deviation; N, population sample size; HDL, high-density lipoprotein; dB, decibel; m/s, meters per second.

**Table S10 P-values for interaction by sex, prediabetes, and type 2 diabetes in the associations of carotid-femoral pulse wave velocity with retinal nerve layer thickness, retinal sensitivity, corneal nerve measures, peripheral nerve conduction velocities and peripheral vibration perception threshold.**

|  | Composite Z-score for retinal nerve layer thickness | Composite Z-score for retinal sensitivity of both eyes | Composite Z-score for corneal nerve measures | Composite Z-score for peripheral NCV | Composite Z-score vibration perception threshold of right and left first toe |
| --- | --- | --- | --- | --- | --- |
| cfPWV, per SD | P-value | P-value | P-value | P-value | P-value |
| Sex | 0.09 | 0.58 | 0.43 | 0.07 | 0.75 |
| Prediabetes | 0.82 | 0.83 | 0.99 | 0.86 | 0.25 |
| Type 2 diabetes | 0.37 | 0.66 | 0.20 | 0.19 | 0.64 |

Variables entered in model: crude + adjusted for age, sex, glucose metabolism status, educational level, mean arterial pressure, waist circumference, alcohol consumption status, smoking status, total cholesterol-to-HDL cholesterol ratio, use of lipid-modifying medication, Dutch Health Diet score and accelerometer-assessed physical activity.

Bold denotes P<0.05.

Abbreviations: HDL, high-density lipoprotein.

**Table S11 Associations of age with composite Z-score for retinal nerve layer thickness, retinal sensitivity, corneal nerve measures, peripheral nerve conduction velocities and peripheral vibration perception threshold.**

|  | | | | | | | | | | | | |
| --- | --- | --- | --- | --- | --- | --- | --- | --- | --- | --- | --- | --- |
|  |  |  | Composite Z-score for retinal nerve layer thickness | | Composite Z-score for retinal sensitivity of both eyes | | Composite Z-score for corneal nerve measures | | Composite Z-score for peripheral NCV | | Composite Z-score vibration perception threshold of right and left first toe | |
|  |  | Model | stβ (95%CI) | N | stβ (95%CI) | N | stβ (95%CI) | N | stβ (95%CI) | N | stβ (95%CI) | N |
| Age, per SD | | 3 | **-0.09 (-0.12 to -0.06)** | 3,131 | **-0.33 (-0.36 to -0.30)** | 4,517 | **-0.09 (-0.13 to -0.06)** | 3,098 | **-0.13 (-0.16 to -0.11)** | 3,709 | **0.38 (0.35 to 0.40)** | 5,119 |

Standardized regression coefficients (stβ) represent the differences in composite Z-score for retinal nerve layer thickness in SD, per SD for age. 1 SD corresponds with 0.8 (unit-less) for composite Z-score for retinal nerve layer thickness, 2.0 dB for retinal sensitivity, 0.9 (unit-less) for composite Z-score for corneal nerve measures, 0.8 (unit-less) for composite Z-score for peripheral nerve conduction velocities, 1.0 (unit-less) for mean of peripheral vibration perception threshold and ≈ 8 – 9 years for age.

Bold denotes P<0.05.

Variables entered in model: crude + adjusted for age, sex, glucose metabolism status, educational level, mean arterial pressure, waist circumference, alcohol consumption status, smoking status, total cholesterol-to-HDL cholesterol ratio, use of lipid-modifying medication, Dutch Health Diet score and accelerometer-assessed physical activity.

For retinal nerve layer thickness, in the fully adjusted model, we compared stβ of cfPWV (-0.04 SD) with stβ for age (-0.09 SD) to calculate the impact of having a 1 SD increase in cfPWV in additional years [calculated as stβ (cfPWV)/ stβ (age) * the number of years per SD]. Having a 1 SD increase in cfPWV corresponds approximately with **4 years of aging**.

For retinal sensitivity, in the fully adjusted model, we compared stβ of cfPWV (-0.06 SD) with stβ for age (-0.33 SD) to calculate the impact of having a 1 SD increase in cfPWV in additional years [calculated as stβ (cfPWV)/ stβ (age) * the number of years per SD]. Having a 1 SD increase in cfPWV corresponds approximately with **2 years of aging.**

For corneal nerve measures, in the fully adjusted model, we compared stβ of cfPWV (-0.05 SD) with stβ for age (-0.09 SD) to calculate the impact of having a 1 SD increase in cfPWV in additional years [calculated as stβ (cfPWV)/ stβ (age) * the number of years per SD]. Having a 1 SD increase in cfPWV corresponds approximately with **5 years of aging**.

For peripheral nerve conduction velocity, in the fully adjusted model, we compared stβ of cfPWV (-0.05 SD) with stβ for age (-0.13 SD) to calculate the impact of having a 1 SD increase in cfPWV in additional years [calculated as stβ (cfPWV)/ stβ (age) * the number of years per SD]. Having a 1 SD increase in cfPWV corresponds approximately with **3 years of aging.**

For peripheral vibration perception threshold, in the fully adjusted model, we compared stβ of cfPWV (0.05 SD) with stβ for age (0.38 SD) to calculate the impact of having a 1 SD increase in cfPWV in additional years [calculated as stβ (cfPWV)/ stβ (age) * the number of years per SD]. Having a 1 SD increase in cfPWV corresponds approximately with **1 year of aging.**

Abbreviations: stβ, standardized beta; CI, confidence interval; SD, standard deviation ; HDL, high-density lipoprotein; cfPWV, carotid-femoral pulse wave velocity.

|  | N = 3,709 |  | Peroneal nerve amplitude | Tibial nerve amplitude | Sural nerve amplitude |
| --- | --- | --- | --- | --- | --- |
|  |  |  | stβ (95% CI) | stβ (95% CI) | stβ (95% CI) |
| Age, per SD |  |  | **-0.21 (-0.25 to -0.18)** | **-0.25 (-0.29 to -0.22)** | **-0.24 (-0.27 to -0.20)** |

**Table S12 Associations of age with peripheral nerve amplitudes.**

Standardized regression coefficients (stβ) represent the differences in Composite Z-score for peripheral nerve conduction velocities in SD, per SD for age. 1 SD corresponds with 2.01 mV for peroneal nerve amplitude, 4.06 mV for tibial nerve amplitude, 6.42 µV for sural nerve amplitude and 8.6 years for age.

Bold denotes P<0.05.

Variables entered in model: crude + adjusted for age, sex, glucose metabolism status, educational level, mean arterial pressure, waist circumference, alcohol consumption status, smoking status, total cholesterol-to-HDL cholesterol ratio, use of lipid-modifying medication, Dutch Health Diet score and accelerometer-assessed physical activity.

In the fully adjusted model, for the tibial nerve amplitude we compared stβ of cfPWV (-0.05 SD) with stβ for age (-0.25 SD) to calculate the impact of having a 1 SD increase in cfPWV in additional years [calculated as stβ (cfPWV)/ stβ (age) * the number of years per SD]. Having a 1 SD increase in cfPWV corresponds approximately with **2 years of aging.**

Abbreviations: stβ, standardized beta; CI, confidence interval; SD, standard deviation ; HDL, high-density lipoprotein; cfPWV, carotid-femoral pulse wave velocity; mV, millivolts; µV, microvolts.
